# Nanopeptide C-I20 as a novel feed additive effectively alleviates detrimental impacts of soybean meal on mandarin fish by improving the intestinal mucosal barrier

**DOI:** 10.3389/fimmu.2023.1197767

**Published:** 2023-06-26

**Authors:** Xingchen Huo, Qiwei Zhang, Jiao Chang, Gang Yang, Shan He, Chunrong Yang, Xufang Liang, Yongan Zhang, Jianguo Su

**Affiliations:** ^1^Hubei Hongshan Laboratory, College of Fisheries, Huazhong Agricultural University, Wuhan, China; ^2^Laboratory for Marine Biology and Biotechnology, Pilot National Laboratory for Marine Science and Technology, Qingdao, China; ^3^College of Veterinary Medicine, Huazhong Agricultural University, Wuhan, China

**Keywords:** antimicrobial peptide, nanopeptide, soybean meal, mucosal barrier, growth performance, fillet quality

## Abstract

Antibacterial peptide has been widely developed in cultivation industry as feed additives. However, its functions in reducing the detrimental impacts of soybean meal (SM) remain unknown. In this study, we prepared nano antibacterial peptide CMCS-gcIFN-20H (C-I20) with excellent sustained-release and anti-enzymolysis, and fed mandarin fish (*Siniperca chuatsi*) with a SM diet supplemented with different levels of C-I20 (320, 160, 80, 40, 0 mg/Kg) for 10 weeks. 160 mg/Kg C-I20 treatment significantly improved the final body weight, weight gain rate and crude protein content of mandarin fish and reduced feed conversion ratio. 160 mg/Kg C-I20-fed fish maintained appropriate goblet cells number and mucin thickness, as well as improved villus length, intestinal cross-sectional area. Based on these advantageous physiological changes, 160 mg/Kg C-I20 treatment effectively reduced multi-type tissue (liver, trunk kidney, head kidney and spleen) injury. The addition of C-I20 did not change the muscle composition and muscle amino acids composition. Interestingly, dietary 160 mg/Kg C-I20 supplementation prevented the reduction in myofiber diameter and change in muscle texture, and effectively increased polyunsaturated fatty acids (especially DHA + EPA) in muscle. In conclusion, dietary C-I20 in a reasonable concentration supplementation effectively alleviates the negative effects of SM by improving the intestinal mucosal barrier. The application of nanopeptide C-I20 is a prospectively novel strategy for promoting aquaculture development.

## Highlights

1. Nanopeptide C-I20 has the excellent effects for slow-release and anti-enzymolysis.
2. Nanopeptide C-I20 effectively reduces the negative effects of soybean meal.
3. C-I20 maintains suitable intestinal mucosal barrier and intestine morphology.
4. C-I20 significantly reduces multi-type tissue injury.
5. C-I20-fed improves the growth performance and fillet quality traits.

## 1 Introduction

With the expansion of aquaculture, the feed industry has experienced remarkable growth in recent years. Fish meal (FM) has earned a reputation as a top-notch protein source for aquatic animal feed because of its high protein content, good palatability, easy absorption and balanced composition of essential amino acids (1). The supply of fish meal, however, cannot keep up with the demand for aquaculture due to the intensive rapid development of aquaculture, which causes the price of fish meal and feed to continuously increase. At present, the addition of plant protein (such as soybean protein and cottonseed protein) alleviates the pressure of insufficient supply of FM, which offers a wide source, low price and easy production compared with animal protein and microbial protein, becoming the ideal replacement for some fish meal (2, 3). However, the fillet quality traits and development rate of breeding objects will be adversely impacted by the presence of anti-nutritional elements (such as saponins, antivitamin factor and phytic acid) and amino acid imbalance in plant protein (4). A previous study reported that soybean meal (SM) may lead to an imbalance in the oxidation/antioxidant system in farmed animals and enhanced hardness of farmed animal muscle (5). In addition, prolonged feeding of SM to fish induced apoptosis and elevated inflammation (6, 7). Cell apoptosis and elevated inflammation also enhanced the tissue damage and hardness of farmed animal muscle (8). A study in grass carp revealed that the application of soybean meal and rapeseed meal (RM) reduced muscle lipid, eicosapentaenoic acid (EPA), docosahexaenoic acid (DHA) and n-3 PUFA contents compared to fishmeal (FM), and SM significantly improved muscle hardness (9).

Antibacterial peptides (AMPs) are short peptides with cationic and amphipathic characteristics, and they widely exist in various life forms from microorganisms to humans (10). AMPs have received extensive research as a bioactive peptide and has been used as a feed additive (11). It has been demonstrated that several AMPs, such as antimicrobial peptides A3 and P5, colicin E1, cecropin AD and lactoferricin, have positive impacts on growth performance, intestinal morphology, antioxidant capacity and fecal microflora (12, 13). It has been reported that some antimicrobial peptides have significant effects on the regulation of the intestinal mucosal barrier (14, 15). The intestinal epithelium provides a selective permeable intestinal mucosal barrier as it allows the passage of water, electrolytes and dietary nutrients but prevents a detrimental invasion of antinutritional factors (14). In addition, antimicrobial peptides, an important active component of compound feed, play an important role in preventing inflammation (11, 12). Therefore, we speculate that antimicrobial peptides with the ability to inhibit inflammation and regulate the intestinal mucosal barrier can improve the growth performance and muscle quality of cultured fish. When AMPs interact with the complex physiological environment, their effectiveness is quickly diminished. The bioavailability of AMPs is greatly reduced by biological digestion, which diminishes their effectiveness (10). Recently, carboxylmethyl chitosan (CMCS) have been applied as the polymer materials to inhibit the degradation of bioactive peptide (16, 17). In our previous study, we identified a AMP gcIFN-20H (I20) with high efficiency and broad-spectrum bactericidal ability from grass carp type I interferon (IFN1) (18). We conjugated gcIFN-20H and CMCS to develop a novel AMP reagent CMCS-gcIFN-20H (CMCS-20H), and found CMCS-20H can retard degradation, prevent bacterial infection, improve intestinal mucosal barrier and regulate intestinal flora (19). Therefore, dietary nanopeptide CMCS-20H supplementation great potential to alleviated negative effect caused by soy protein in fish, thus improving the growth performance and fillets quality of cultured fishes.

In previous studies, we used shake flasks for yeast expression, but only a small amount of gcIFN-20H was obtained. In order to further meet the industrial demand, high expression strains were put into 10 L fermenters for high-density culture to obtain a large number of gcIFN-20H. CMCS-20H and C-I20 were prepared on the same principle, and the same raw materials and ratios were added during the preparation process. Due to the different expression methods, we named the nanopeptide CMCS-gcIFN-20H prepared by this method C-I20 to distinguish CMCS-20H.

Mandarin fish (*Siniperca chuatsi*) is one of the most popular cultured fish in China because of delicious taste, abundant nutrition and high market value (20). Chinese perch fed on artificial diet have been successfully domesticated through special procedures (20, 21). However, the series of side effects caused by excessive substitution of fish meal with soybean meal have not been fully resolved. Hence, the mandarin fish was employed as a representative experimental animal to test the efficacy of nanopeptide C-I20. In this study, we found that C-I20 exhibited excellent sustained-release and anti-enzymolysis in a simulated physiological environment. Dietary 160 mg/Kg C-I20 supplementation dramatically reduce inflammation of various internal organs, and improve intestinal mucosal barrier, intestinal morphology, growth performance, fillets quality and fillets nutritive value. According to our knowledge, this study marks the first time antimicrobial peptides agent have been employed to improve growth performance and fillet quality traits by diminish the negative effects of plant proteins in aquaculture.

## 2 Materials and methods

### 2.1 Preparation of nanopeptide CMCS-gcIFN-20H (C-I20)

#### 2.1.1 Yeast high cell density expression and gcIFN-20H purification

*Pichia pastoris* GS115 expressing gcIFN-20H were preserved in our laboratory (22). The selected recombinant GS115 strain was inoculated into a flask containing 100 mL of yeast peptone dextrose (YPD) media (1% yeast extract, 2% peptone and 2% dextrose), followed by incubation at 28°C, 250 rpm. When OD600 reached about 8, the cell cultures (100 mL) were transferred into 6 L BSM medium base (Haibo, Qingdao, China) media loaded in a fermenter (10 L) for high cell density cultivation (BIOTECH-10BS, Baoxing, Shanghai, China). The glycerol was supplied as carbon source for cell growth, followed by I20 expression being induced with methanol. After 96 hours of methanol induction, the supernatant was obtained by centrifugation at 12,000×g for 2 min. I20 peptide in supernatant was purified by His-tag Protein Purification Kit (Beyotime, Shanghai, China).

#### 2.1.2 Conjugating gcIFN-20H and CMCS

Nanopeptide CMCS-gcIFN-20H (C-I20) was prepared according to the previously described ionic gelation method (22). Briefly, I20 (50 mg/mL, pH = 7.4) and CMCS (50 mg/mL, pH = 7.4) solutions were mixed for 30 min under magnetic stirring, collected *via* centrifugation at 12,000 rpm at 4°C for 1 h, washed with sterile ultrapure water thrice, then resuspended in distilled water using a probe sonication (pulse on, 3.0 s; pulse off, 2.0 s; 1 min/cycle; power 130 W) for storage. FITC-C-I20 nanoparticles were also prepared according to the above method. FITC-labeled gcIFN-20H (FITC-I20) peptides were synthesized by GenScript Biotech Corporation (Nanjing, China).

#### 2.1.3 Microscopy and dynamic light scattering assay

The morphology of C-I20 was observed by H-7650 transmission electron microscopy (TEM, HITACHI, Tokyo, Japan) and N-sim structured illumination microscopy (SIM, Nikon, Tokyo, Japan). Dissolve C-I20 in double distilled water (pH = 7.0) to form 1mg/ml C-I20 solution. The particle size distribution and zeta potential of C-I20 were determined by dynamic light scattering using a Malvern Nano-ZS 90 laser particle size analyzer (Malvern Instruments, UK) at a detector angle of 90°, 670 nm, and 25°C.

#### 2.1.4 Drug loading efficiency and release efficiency assay

The amount of I20 carried on CMCS nanoparticles was determined by measuring the amount of protein remaining in supernatant by BCA protein assay. In release PBS medium (pH = 2.0 or pH = 10.0), *in vitro* release profile of C-I20 was detected at 25°C for 20 h. 1 mL C-I20 (1 mg/mL) were placed into EP tubes with 1 mL of release medium at 100 rpm. The protein content in supernatant was measured by BCA method at specified time intervals. The LE and RE were calculated according to formulas:


LE=(Wtg−Wng)/Wmg×100%

RE=Wsg/Wng×100%

Where Wtg, Wng and Wmg refer to the weight of total I20, and I20 in supernatant and CMCS respectively (n = 6). Where Wsg and Wng refer to the weight of gcIFN-20H in supernatant and carried respectively (n = 6).

### 2.2 Anti-enzymolysis assays of C-I20 and gcIFN-20H

To determine the anti-enzymolysis effect of C-I20, we employed 10 mL simulated gastric juice (100 μg/mL pepsin, pH = 2.0), and then added C-I20 (final concentration is 5 mg/mL, C-I20 group) or gcIFN-20H (final concentration is 1 mg/mL, gcIFN-20H group), respectively. Take 1 mL of analog solution in gcIFN-20H group and C-I20 group at different times (0.5 h, 1 h, 1.5 h, 2 h). The analog solution in gcIFN-20H group was directly detected by SDS-PAGE assay. The C-I20 was collected *via* centrifugation at 12,000 rpm at 4°C for 1 h. The collected C-I20 was added to the release solution and allowed to stand for 12 h after resuspended by probe sonication. The CMCS and residual C-I20 in release solution was removed by centrifugation, and the residual solution was detected by SDS-PAGE assay and ImageJ software V1.8.0 (NIH, Bethesda, U.S.A).

### 2.3 Diet and feeding trial

#### 2.3.1 Experimental fish and ethical statement

Mandarin fish (90 day ages, n > 1000, weight = 30.73 ± 0.31 g, body length = 13.85 ± 0.25 cm) was obtained from the Chinese Perch Research Center of Huazhong Agricultural University (Wuhan, China) and reared in a recirculating water system (250 L each tank, water temperature of 21 ± 0.5°C, dissolved oxygen of 9 ± 0.5 mg/L, pH of 7.2 ± 0.2, ammonia<0.1 mg/L, nitrite<0.1 mg/L, and salinity<0.1‰).

All the animal experiments were approved by the Ethical Committee on Animal Research in Huazhong Agricultural University (ID Number: HZAUFI-2022-0031). All the efforts were made to minimize animal suffering. Before handling, fish were anesthetized using 3-Aminobenzoic acid ethyl ester methanesulfonate (140 mg/L, Solarbio, Beijing, China).

#### 2.3.2 Experimental diets

Formulation of the semi-purified basal diet was modified according to our previous research, and was shown in **Table S1**. Fish meal, casein, and soybean meal were used as the dietary protein sources. Fish oil were used as dietary lipid sources. Two control diets were designed as follows: the positive control diet (FM group) was prepared with 48% of dietary FM plus 0% of SM; The negative control diet (control group) was prepared with 30% of dietary FM plus 18% of SM. Based on the negative control diet, C-I20 was added to provide graded levels of antimicrobial peptide at final concentration of 40, 80, 160 and 320 mg/Kg. The diets were prepared separately and extruded into 3 mm pellets as performed regularly in our laboratory. The prepared pellets were stored in a refrigerator at -80°C until needed for the feeding trial.

#### 2.3.3 Feeding management

The water source in the aquaculture system was from groundwater with relatively stable water temperature. Each tank (L 1.2 m × W 0.6 m × H 1.0 m with water depth of 0.5 m) was equipped with a 50W air pump providing continuous aeration to each tank, and freshwater was supplied daily from 8 a.m. to 6 p.m. at a speed of about 20 L/min. During the feeding trial, the water temperature was measured three times from morning to evening, and maintained at 21 ± 0.5°C. During the experimental period, the water quality parameters including temperature, pH and dissolved oxygen were measured daily. Normally, we change the water once a week and the amount of changed water is about 20%, but as long as the quality index is not up to the standard we will change the water in time. Before the feeding trial, a total of 540 fish were selected and weighed individually to obtain initial body weight (IBW). The fish were fasted for 24 h and randomly stocked into 18 automatically aerating and circulating tanks. Each treatment contained three replicates with 30 fish per tank. Each cage was equipped with a disc of 100 cm in the bottom to collect uneaten food and feces. During the 10 weeks feeding trial, each of the six experimental diets was fed to fish two times daily (8: 00 and 18: 00). Fish were fed to satiation, and uneaten food on the disc was carefully removed by siphoning after fed 40 min, which was appropriately adjusted according to the weather, temperature and feeding behavior to ensure the fish were fed to satiation.

### 2.4 Determination of growth performance, feed efficiency and body composition

At the end of the feeding trial, the fish were deprived of diets for 12 h, and then counted the individual fish body weight in each tank to calculate the final body weight (FBW). Four fish in each tank (twelve fish in each group) were sampled for measurement of morphological parameters including visceral weight and liver weight for the analysis of growth performance. Weight gain rate (WGR), feed conversion ratio (FCR), survival rate, viscerosomatic index (VSI), hepatosomatic index (HSI) were calculated by using the following formulae:


WGR(%)=100×(final weight−initial weight)/initial weight

FCR=Feed intake amount/(Final fish weight−Initial fish weight)

Survival rate(%)=100×(final number of fish/initial number of fish)

VSI(%)=100×(final visceral weight/final body weight)

HSI(%)=100×(final liver weight/final body weight)

Proximate composition of whole fish was analyzed by referring to a previous study (20). Four fish in each tank (twelve fish in each group) were randomly collected for body composition analysis. The moisture content was detected by drying for 4 h at 105°C in a drying oven. The content of crude lipids was evaluated by ether-extraction with Soxtec System HT (SE-A6, Alvah, China). After acid digestion, the content of crude protein (N × 6.25) was determined in the Kjeltec system with the K8400 Kjeltec Analyzer (Fossana Lyticab, Sweden). The content of ash was analyzed in a muffle furnace for 6 h at 550°C.

### 2.5 Serum biochemistry index assay and histological examination

After feeding trial, twelve fish blood samples in each group were collected for serum biochemistry index firstly, and then the twelve fish tissue (intestine, liver, trunk kidney, spleen, head kidney and muscle) in each group were collected for histological analysis.

Blood samples were collected from the caudal vein and placed for 4 h at 25°C. After centrifugation (4,000 rpm/min, 5 min) at 4°C, the serum samples were collected and stored at -80°C. The serum biochemical indexes of alanine transaminase (ALT), alkaline phosphatase (ALP) and aspartate aminotransferase (AST) were examined by the commercial kits (Nanjing Jiancheng Bioengineering Institute, Nanjing, China).

For the histology and design-based stereology studies, mid intestine samples were collected as described in our previous publications. Periodic Acid-Schiff (PAS) staining was used to detect goblet cells and mucus layer thickness in intestinal villi. The intestine tissue was fixed in Methanol-Carnoy’s fixative (Solarbio, Beijing, China) at 4°C for 2 h, then transferred to 100% ethanol for storage. Fixed intestinal tissues were washed with distilled water for 5 min and embedded in paraffin. 4 μm sections were deparaffinized and then stained with PAS.

For morphology and pathology observation, the tissues (mid intestine, liver, trunk kidney, spleen, head kidney and muscle) were fixed in 10% neutral buffered formalin for 24 h, and embedded by paraffin. 4 μm sections were mounted on 3-aminopropyltriethoxysilane-coated slides. Following the deparaffinization in xylene, sections were rehydrated, stained with hematoxylin and eosin (H&E, Solarbio, Beijing, China), and mounted with neutral gum, then the images were captured.

For the Oil Red O staining of tissues, the muscles fixed in 4% perfluoroalkyl resin were produced to frozen section and then treated with 60% isopropanol for 2 min. Following this, the sections were immersed with 0.3% Oil Red O (Solarbio, Beijing, China) for 20 min at room temperature. Subsequently, Oil Red O was removed, the sections were washed with 60% isopropanol for one time and again with PBS for twice. After hematoxylin re-staining, the sections were observed under a microscope.

All images were captured using an Eclipse Ti-SR microscope with a DS-U3 Image-Pro system (Nikon, Tokyo, Japan). Moreover, three fields of view were randomly selected in each slice (three view per slice, one slice per fish, 12 fish per treatment) for per detection analysis (n = 12). The value of each sample used for statistical analysis is the average value of statistics under three random fields of vision. The morphological characteristics and pathological changes of tissues were observed by CaseViewer software 2.4 (IBM, New York, U.S.A). The analysis results were calculated by ImageJ software V1.8.0 (NIH, Bethesda, U.S.A) and CaseViewer software 2.4.

### 2.6 Texture profile analysis (TPA) and muscle composition analysis

Four fish in each tank (twelve fish in each group) anesthetized and dissected to obtain white muscle samples for analysis of flesh texture parameters (hardness, springiness, and chewiness). Muscle samples were cut into thick sections (1 cm × 0.5 cm × 0.5 cm) and TPA analysis was performed immediately. TPA of muscle was determined by the texture analyzer (Stable Micro Systems, England). The measurement parameters were as follows: 5 mm for return distance; 1 mm/s for return speed; and 2 g for contact force. The remaining muscles after TPA were used for muscle composition analysis. The composition analysis of muscle is consistent with the methods and parameters of the above body composition analysis.

### 2.7 Amino acid content and fatty acid analysis

For amino acids and fatty acids composition analyses, twelve fish flesh samples per group were collected and four flesh samples were mixed as a pooled sample. The contents of amino acids in the experimental diets (n = 3) and muscle (n = 3) were analyzed with Amino Acids Automatism Analyzer (L-8900, Hitachi, Tokyo, Japan) after acid hydrolysis at Hubei Feed Quality Station of Analysis and Supervisor (Wuhan, China). Amino acid compositions of the tested diets was shown in **Table S2**. The fatty acid methyl esters (FAME) were analysed on a gas chromatograph (Thermo Finnigan Co., San Jose, U.S.A) equipped with a capillary column (30 m × 32 mm). The details on the method has been described in previous method (23). Fatty acid composition of the tested diets was shown in **Table S3**.

### 2.8 Statistical analysis

The results were expressed as the means ± standard deviation (SD) and all statistical analysis were done using SPSS 26.0 package (IBM, New York, USA). The difference in anti-enzymolysis assays was analyzed by one-way analysis of variance (ANOVA) in SPSS version 11.0 (SPSS Inc., Chicago, IL, USA). Significance (p-value) is indicated as: *(p< 0.05), **(p< 0.01) and ***(p< 0.001). The experimental data of six groups were subjected to the Kruskal-Wallis test followed by Dunn’s multiple comparison (with Bonferroni adjustment) to identify the significance (p< 0.05). Values with the same letter superscripts represent no significant difference, while with different letter superscripts represent significant differences. The redundancy analysis was processed by Canoco5 software. The protection rate data were analyzed by Mantel-Cox test (ns > 0.05, *p< 0.05, **p< 0.01).

## 3 Results

### 3.1 Nanoparticle C-I20 possesses sustained-release and excellent anti-enzymolysis

**Figure 1:**
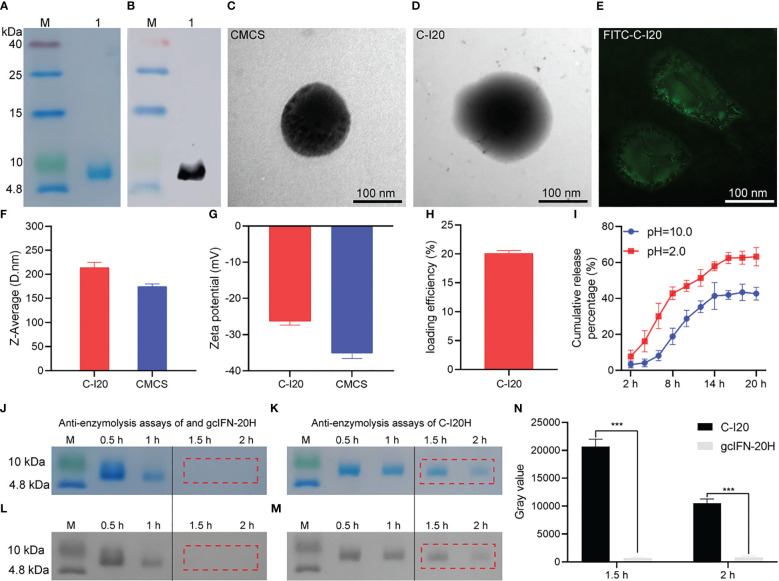
The detection of preparation, characterization, release efficiency, antidegradation efficacy of nanopeptide C-I20. SDS-PAGE and WB analyses of gcIFN-20H **(A, B)**. Lane M: protein marker; Lane 1: purified gcIFN-20H. Transmission electron micrograph of CMCS nanoparticles **(C)**. Transmission electron micrograph of C-I20 nanoparticles **(D)**. Structured illumination microscopy micrograph of FITC-C-I20 nanoparticles **(E)**. Nanoparticle size analysis **(F)**. Zeta potential analysis **(G)**. Loading efficiency analysis **(H)**. *In vitro* release curve of nanopeptide C-I20 in different pH solutions (2.0 and 10.0) **(I)**. Anti-pepsin degradation analysis **(J, K)**. SDS-PAGE gray value analysis **(L, M)**. Quantitative analysis of gray value **(N)**. The difference in anti-enzymolysis assays was analyzed by one-way analysis of variance (ANOVA) in SPSS version 11.0 (SPSS Inc., Chicago, IL, USA). Significance (p-value) is indicated as: ***(p< 0.001). Data are presented as means ± SD (n = 6).

gcIFN-20H was purified and analyzed by SDS-PAGE (**Figure 1A**). WB verified that gcIFN-20H was the main protein band on the SDS-PAGE gel (**Figure 1B**). By TEM observation, the CMCS was dark microsphere (**Figure 1C**), and protein shadow presented on the surface of CMCS after gcIFN-20H was loaded by CMCS (**Figure 1D**). According to SIM investigation, the FITC-gcIFN-20H loaded onto the surface of CMCS to form green fluorescent particles (**Figure 1E**). The particle sizes of CMCS and C-I20 were 174.5 ± 5.3 nm and 213.8 ± 11.1 nm respectively (**Figure 1F**), and the zeta potentials of CMCS and C-I20 were -35.1 ± 1.5 mV and -26.3 ± 1.1 mV respectively (**Figure 1G**). LE of C-I20 was 20.2 ± 0.7% (**Figure 1H**). C-I20 released more gcIFN-20H in acidic condition (pH = 2.0) than in alkaline condition (pH = 10.0) (**Figure 1I**). According to SDS-PAGE and ImageJ analysis, pepsin completely digested gcIFN-20H within 1 to 2 hours (**Figures 1J, L**), but gcIFN-20H loaded onto the surface of C-I20 was not completely digested by pepsin within 2 hours (**Figures 1K, M**). In the statistical analysis of gray value, the gray value of C-I20 group was significantly higher than that of gcIFN-20H group in 1.5 h and 2 h (**Figure 1N**). These results indicate that nanopeptide C-I20 is a spherical nanoparticle with features of uniform particle size, sustained release and anti-enzymolysis.

### 3.2 The 160 mg/Kg C-I20 treatment significantly increases the growth performance

No deaths were reported throughout the feeding period, and there was no discernible difference in IBW between the groups, as indicated in **Table 1**. Fish fed 160 mg/kg C-I20 diet exhibited the highest FBW, WG and lowest FCR of all the C-I20 supplementation groups and control group. No discernible variation in VSI and HSI was obtained in all of the experimental diets. Fish in 160 mg/kg C-I20 group exhibited the highest amount of crude protein of all the C-I20 supplementation groups and control group. There was no significant difference in the crude protein amount of between FM group and 160 mg/Kg group. In all of the experimental diets, there was no discernible variation in the amounts of moisture, crude lipid or ash. In the growth parameter and body composition, there was no significant difference between FM group and 160 mg/kg group. Generally speaking, these data suggest that 160 mg/Kg C-I20 supplementation mainly alleviated the SM-caused increase of feed coefficient and accordingly increased the weight gain rate and crude protein deposition.

**Table 1:** Growth performance, feed utilization and body composition of mandarin fish fed different levels of C-I20 diets for 10 weeks.

Item	Group					
	FM	320 mg/Kg	160 mg/Kg	80 mg/Kg	40 mg/Kg	Control
SR (%)	100	100	100	100	100	100
IBW (g)	31.06± 0.99^a^	30.51± 0.49^a^	30.83± 0.37^a^	31.26± 0.45^a^	30.88± 0.51^a^	30.76± 0.54^a^
FBW (g)	78.49± 2.36	63.72± 3.81^b^	74.49± 4.18^c^	55.75± 4.27^a^	53.11± 5.12^a^	54.23± 2.83^a^
WGR (%)	152.74 ± 7.37^d^	108.83 ± 12.51^c^	141.75 ± 16.17^d^	78.42 ± 14.22^b^	71.86 ± 14.71^a^	76.45 ± 12.03^b^
FCR	1.26± 0.50^a^	1.45± 0.08^b^	1.29± 0.05^a^	1.54± 0.04^bc^	1.60± 0.02^c^	1.54± 0.03b^c^
VSI (%)	13.18± 0.20^a^	13.27± 0.12^a^	13.55± 0.55^a^	12.66± 0.32^a^	13.25± 0.17^a^	12.98± 0.14^a^
HSI (%)	2.05± 0.25^a^	2.14± 0.23^a^	2.09± 0.33^a^	1.93± 0.28^a^	1.95± 0.19^a^	2.01± 0.15^a^
Moisture (%)	75.68± 0.62^a^	75.23± 0.42^a^	75.71± 0.75^a^	75.54± 0.35^a^	75.28± 0.77^a^	75.47± 0.84^a^
CP (%)	18.85± 0.28^b^	17.23± 0.12^a^	18.55± 0.35^b^	17.02± 0.23^a^	16.88± 0.41^a^	17.25± 0.24^a^
CL (%)	2.25± 0.24^a^	2.32± 0.22^a^	2.24± 0.51^a^	2.33± 0.14^a^	2.09± 0.31^a^	2.19± 0.36^a^
Ash (%)	4.89± 0.62^a^	5.13± 0.64^a^	4.82± 0.92^a^	4.94± 0.35^a^	5.07± 0.74^a^	4.86± 0.45^a^

Values in the same row with different superscripts indicate significant differences (P< 0.05). SR, survival rate; IBW, initial body weight; FBW, final body weight; WGR, weight gain rate; FCR, feed conversion ratio; VSI, viscerosomatic index; HSI, hepatosomatic index; CP, crude protein; CL, crude lipid.

### 3.3 The 160 mg/Kg C-I20 treatment effectively improves intestinal morphology

**Figure 2:**
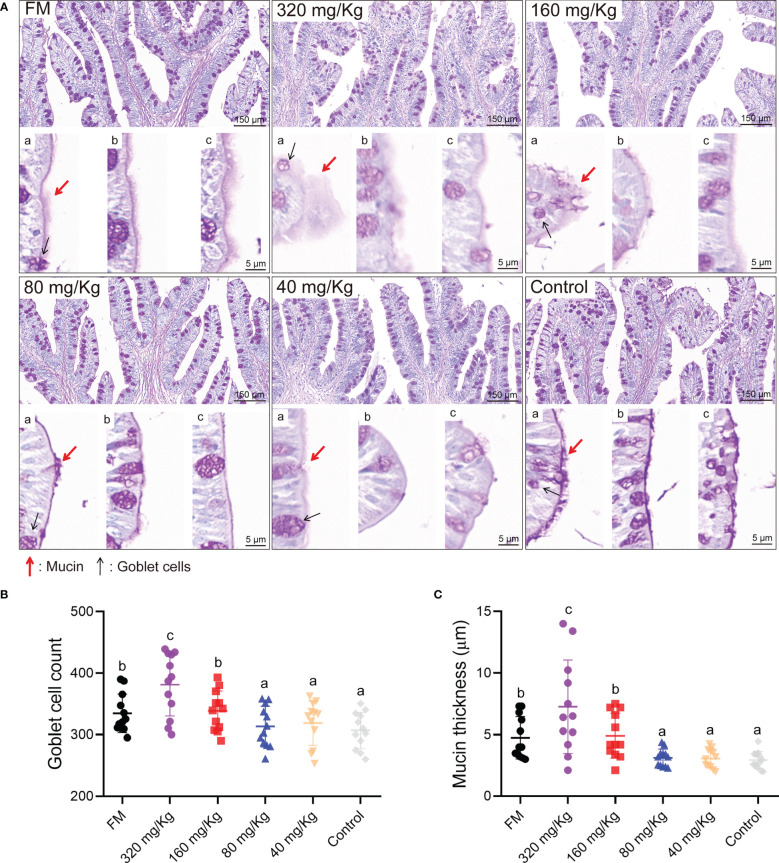
The intestinal mucosal barrier of mandarin fish fed diets containing various levels of C-I20. The PAS staining microscopic image of intestinal goblet cells and mucins **(A)**. Mucin thickness and goblet cell morphology were displayed by different visual fields (a, b, and c). The mucin was marked with red arrows. The goblet cells were marked with black arrows. The number of goblet cells **(B)**. The thickness of mucin **(C)**. Twelve fields were equally and randomly selected in four section for goblet cell count and mucin thickness analysis. Different superscript letters in each group denote significance variations suggested by the Kruskal–Wallis statistics at 95% of significance, followed by the Dunn test with Bonferroni adjustment as the *post hoc* test (p< 0.05). Data are presented as means ± SD (n = 12).

The intestinal goblet cell number and mucin thickness were analyzed by PAS staining. Overall, the number of goblet cells and mucin thickness increased with increasing C-I20 dose as measured by PAS staining (**Figure 2A**). By statistical analysis, the number of goblet cells and mucins on intestinal villi increased significantly in the 320 mg/Kg group compared with the other groups (**Figures 2B, C**). The number of goblet cells and mucins on intestinal villi increased significantly in the 160 mg/Kg and FM groups compared with 80 mg/Kg, 40 mg/Kg, and control groups, while there was no significant difference between in 160 mg/Kg group and FM group (**Figures 2B, C**). In conclusion, dietary 320 mg/Kg or 160 mg/Kg C-I20 supplementation effectively maintains sufficient mucin and goblet cells in intestinal villi to resist the adverse effects of SM.

**Figure 3:**
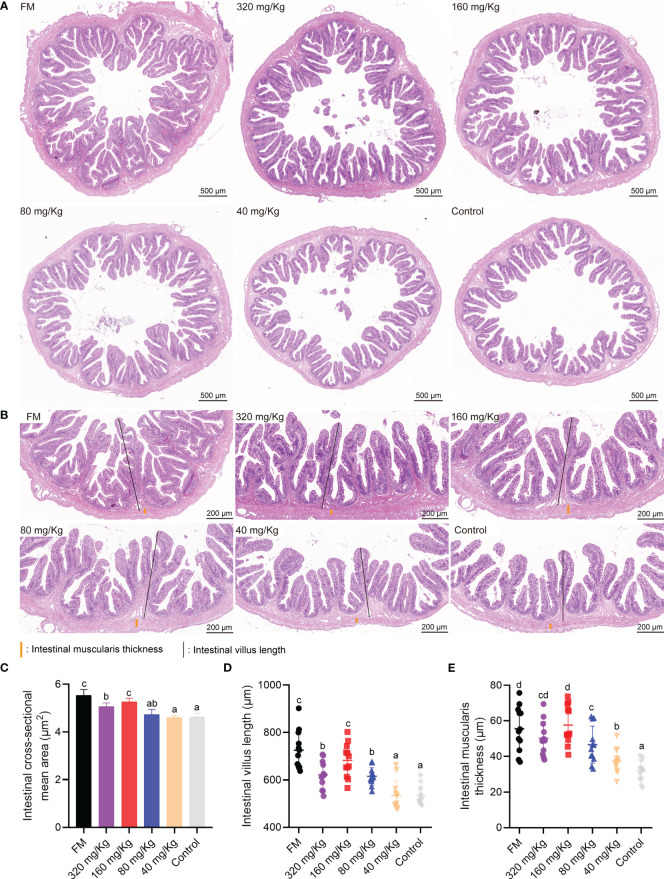
The intestinal physiological structure of mandarin fish fed diets containing various levels of C-I20. A cross section of the intestine is detected by H&E staining **(A)**. The H&E staining microscopic image of intestinal features **(B)** The intestinal muscularis thickness were marked with orange vertical lines. The intestinal villus length was marked with black vertical lines. Detection of intestinal cross-sectional mean area **(C)**, intestinal villus length **(D)** and muscularis thickness **(E)**. Different superscript letters in each group denote significance variations suggested by the Kruskal–Wallis statistics at 95% of significance, followed by the Dunn test with Bonferroni adjustment as the *post hoc* test (p< 0.05). Data are presented as means ± SD (n = 12).

In the intestinal H&E staining section, no tissue lesion was observed in any of the groups (**Figure 3A**). With the increase of C-I20 supplementation dose, the intestinal cross-sectional area tended to increase (**Figure 3A**). The number of intestinal villus length and muscularis thickness increased with increasing C-I20 supplementation dose as measured (**Figure 3B**). Compared to the control group, the mean area of intestinal cross-sectional was remarkably increased in the FM, 320 mg/Kg, and 160 mg/Kg groups (**Figure 3C**). Intestinal villus length was markedly longer in the FM and 160 mg/Kg groups than that in the other groups (**Figure 3D**), and muscularis thickness was markedly thicker in FM, 320 mg/Kg, and 160 mg/Kg groups than that in the other groups (**Figure 3E**). There were no significantly difference in intestinal cross-sectional mean area, villus length, and muscularis thickness between FM group and 160 mg/Kg (**Figures 3C–E**). After SM was used instead of FM, dietary 160 mg/Kg C-I20 supplementation help to increase intestinal cross-sectional mean area, intestinal villi length, and muscularis thickness, which greatly alleviate the negative effects of SM on intestinal morphology.

### 3.4 The 320 mg/Kg or 160 mg/Kg C-I20 treatment significantly reduces multi-type viscera injury

**Figure 4:**
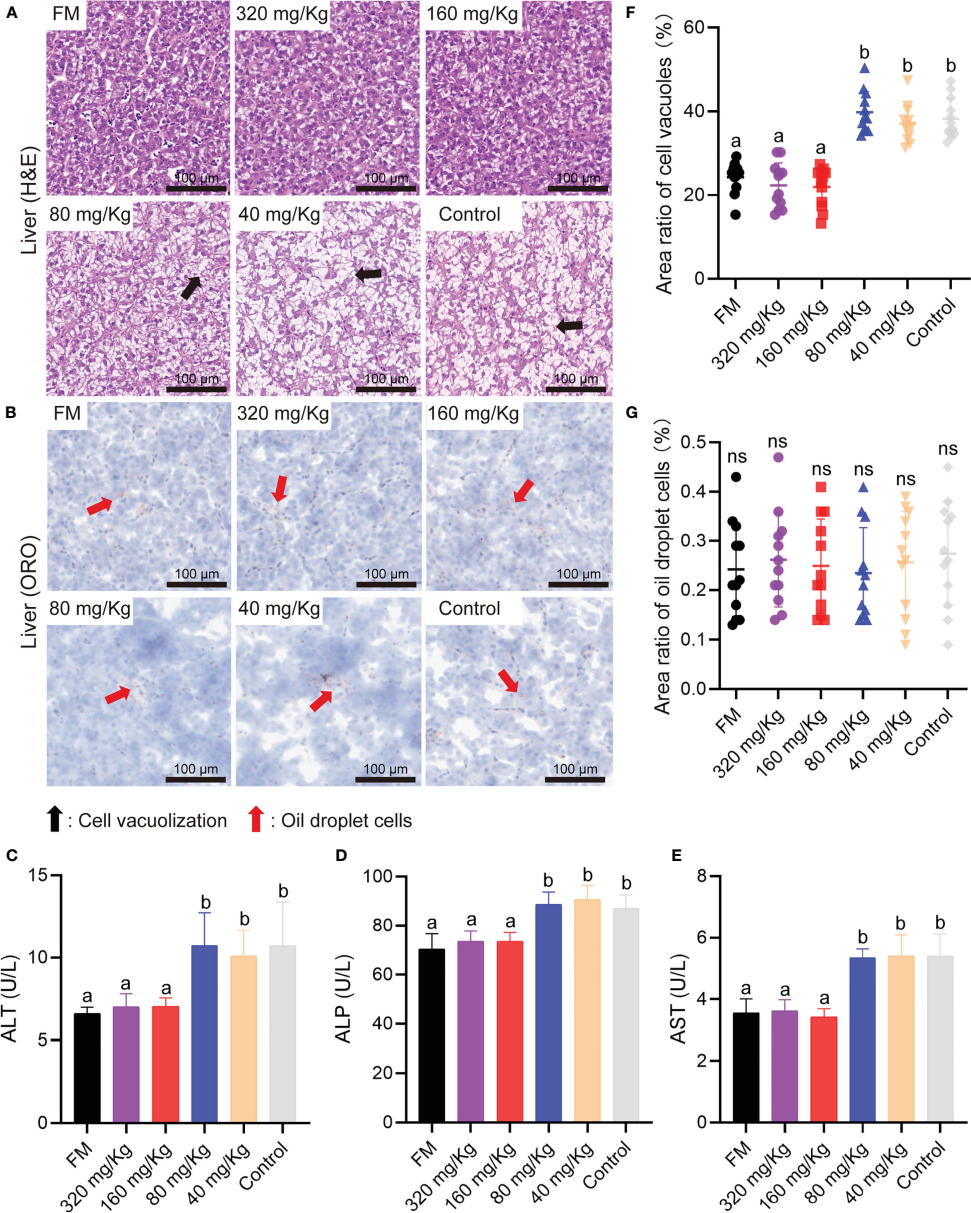
The liver healthy status of mandarin fish fed diets containing various levels of C-I20. Histological analysis of liver by H&E staining **(A)** and oil red O staining **(B)** of the oral groups that received different concentrations of C-I20 nanoparticles. The cell vacuolization was marked with black arrows. The oil droplet cells were marked with red arrows. Liver function related indexes including ALT **(C)**, ALP **(D)**, AST **(E)** reflecting liver function injury was detected by commercial kits (Nanjing Jiancheng Bioengineering Institute, Nanjing, China). Area ratio of cell vacuoles in liver **(F)**. Area ratio of oil droplets cells in liver **(G)**. Different superscript letters in each group denote significant variations suggested by the Kruskal–Wallis statistics at 95% of significant, followed by the Dunn test with Bonferroni adjustment as the *post hoc* test (p< 0.05). Data are presented as means ± SD (n = 12).

To detect the inflammation and health status of the main viscera, liver, trunk kidney, spleen and head kidney were collected on D56 and sectioned with H&E staining or oil red O staining (ORO). First, the metabolism organ of the liver and trunk kidney were examined by histological analysis. The liver H&E staining microimage of the 80 mg/Kg, 40 mg/Kg, and control groups showed the most evident liver vacuole formation, whereas the FM, 320 mg/Kg and 160 mg/Kg groups showed the lesser lesions (**Figure 4A**). There was no discernible difference between either group’s oil droplet cells according to the ORO staining assay (**Figure 4B**). The results of serum activity detection that reflected liver inflammation showed the ALT, ALP and AST activity in FM, 320 mg/Kg and 160 mg/Kg groups was significantly lower than that in 80 mg/Kg, 40 mg/Kg group and control groups (**Figures 4C–E**). By caseview software analysis, the cell vacuoles area ratio in FM, 320 mg/Kg and 160 mg/Kg groups was significantly less than that in 80 mg/Kg, 40 mg/Kg group and control group (**Figure 4F**), while there was no significant difference in the area of the oil droplet cells between any of the groups (**Figure 4G**).

**Figure 5:**
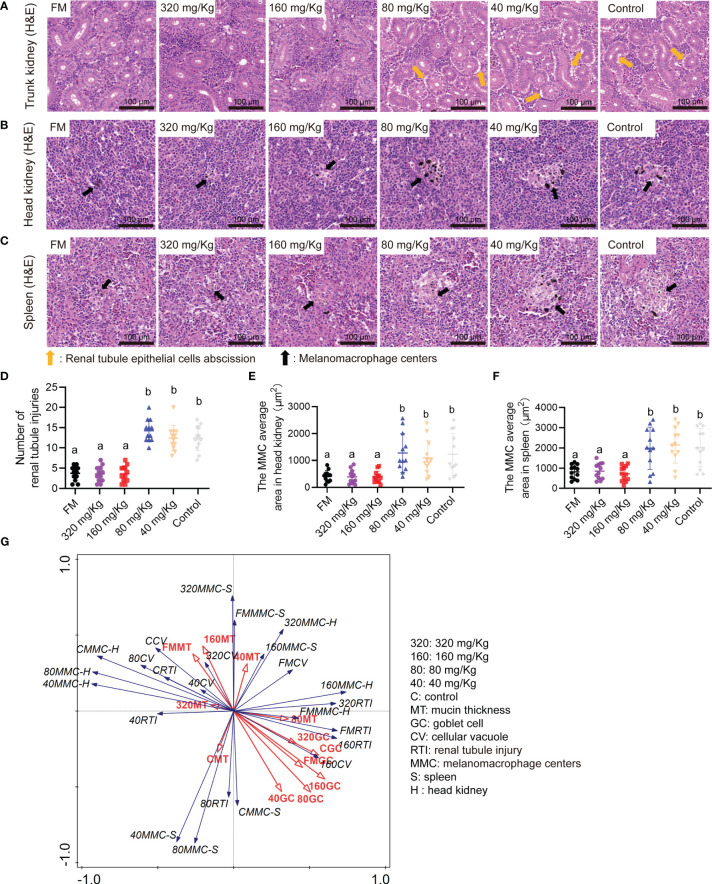
The multi-type tissue healthy status of mandarin fish fed diets containing various levels of C-I20. Histological analysis of trunk kidney by H&E staining of the oral groups that received different concentrations of C-I20 nanoparticles **(A)**. Histological analysis of head kidney **(B)** and spleen **(C)** by H&E staining of the oral groups that received different concentrations of C-I20 nanoparticles. Number of renal tubule injuries in trunk kidney **(D)**. The renal tubule epithelial cells abscission was marked with orange arrows. The melanomacrophage centers (MMC) average area in spleen **(E)** and head kidney **(F)** was calculated by caseview. The melanomacrophage centers were marked with orange arrows. Redundancy analysis (RDA) between the intestinal mucosal barrier and tissues inflammation indicators **(G)**. Different superscript letters in each group denote significant variations suggested by the Kruskal–Wallis statistics at 95% of significant, followed by the Dunn test with Bonferroni adjustment as the *post hoc* test (p< 0.05). Data are presented as means ± SD (n = 12).

By histological analysis, the renal tubule epithelial cells abscission in the trunk kidney were obvious lesions in 80 mg/Kg, 40 mg/Kg groups and control group (**Figure 5A**). The immune organs of the spleen and head kidney were detected by histological analysis. Compared with FM group, the augmented MMC area was observed in spleen and head kidney sections of 80 mg/Kg, 40 mg/Kg groups and control groups (**Figures 5B, C**). In number of injury renal tubule analysis, the number of injury renal tubule in FM, 320 mg/Kg and 160 mg/Kg groups was substantially less than those in the 80 mg/Kg, 40 mg/Kg and control groups (**Figure 5D**). The MMC average area in the FM, 320 mg/Kg and 160 mg/Kg groups was substantially less than that in the 80 mg/Kg, 40 mg/Kg group and control group, according to statistical analysis of the spleen and head kidney (**Figures 5E, F**). Through RDA analysis, we found that the goblet cell number and the mucin thickness in each group were negatively correlated with tissue damage indicators to varying degrees (**Figure 5G**). This indicates that as the number of goblet cells and the thickness of mucin increase, the degree of tissue damage will decrease.

These results show that the dietary 160 mg/Kg C-I20 supplementation reduced vacuole formation, hepatitis serum index (ALT, ALP and AST), renal tubule injury number, and MMC area in head kidney and spleen caused by feeding soybean meal substitute feed.

### 3.5 Dietary 160 mg/Kg C-I20 supplementation effectively maintains fillet texture

**Figure 6:**
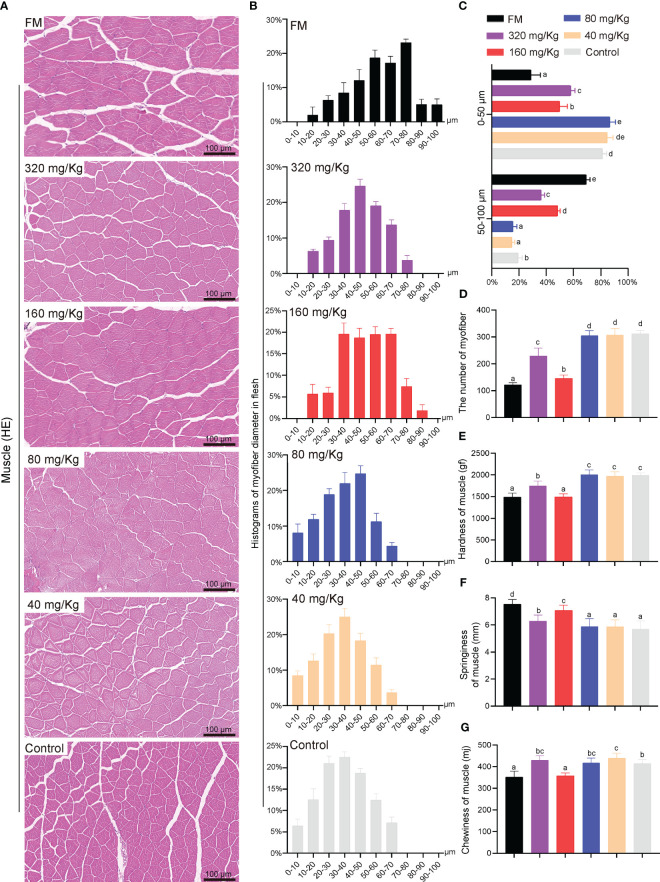
The fillets quality of mandarin fish fed diets containing various levels of C-I20. Histological characteristic analysis of mandarin fish myofiber by H&E staining **(A)**. Histograms of myofiber diameter in flesh of mandarin fish under a field of view of 0.4 mm^2^ area **(B)**. Myofiber diameter distribution in each group **(C)**. Numbers of myofiber under a field of view of 0.4 mm^2^ area **(D)**. The detection of mandarin fish muscle hardness **(E)**, springiness **(F)** and chewiness **(G)**. Different superscript letters in each group denote significant variations suggested by the Kruskal–Wallis statistics at 95% of significance, followed by the Dunn test with Bonferroni adjustment as the *post hoc* test (p< 0.05). Data are presented as means ± SD (n = 12).

Compared to the other groups, the fish in the FM and 160 mg/Kg groups had an increase in myofiber diameter that was evident (**Figure 6A**). More specifically, the fish fed on C-I20 (320 mg/Kg, 80 mg/Kg, 40 mg/Kg and 0 mg/Kg) supplemental diet had higher values of small-diameter myofiber (10-40 μm), but lower values of larger-diameter fibers (50-90 μm) than those fed on 160 mg/Kg C-I20 supplemental feed and FM diet (**Figure 6B**). Only the muscle fibers diameter of the FM group reached 90-100 nm (**Figure 6B**). The fed FM, 320 mg/Kg C-I20 and 160 mg/Kg C-I20 diets fish had lower values of small-diameter myofiber (0-50 μm), but higher values of larger-diameter fibers (50-100 μm) than those fed on the different C-I20 rates (80 mg/Kg, 40 mg/Kg and 0 mg/Kg) (**Figure 6C**). The number of myofibers in FM and 160 mg/Kg groups significantly less than those of the other groups (**Figure 6D**). The mandarin fish in FM and 160 mg/Kg groups had considerably less hardness (**Figure 6E**) and chewiness (**Figure 6G**), but much more springiness (**Figure 6F**) of muscle, compared with the other treatment groups. There was no significant difference between FM group and 160 mg/Kg group in hardness and chewiness (**Figures 6E, G**). In summary, dietary 160 mg/Kg C-I20 prevented the reduction in myofiber diameter and change in fillet texture caused by feeding mandarin fish with soybean protein.

### 3.6 Dietary 160 mg/Kg C-I20 supplementation significantly increases fillets nutritive value

The body composition of muscle of six treatment groups (FM, 320 mg/Kg, 160 mg/Kg, 80 mg/Kg, 40 mg/Kg and Control) are presented in **Table 2**. Among the six treatment groups, there were no significant differences in moisture, crude protein, crude lipid and ash in white muscle (P > 0.05). The ∑EAA, ∑NEAA and ∑FAA values in the other groups (320, 160, 80, 40, 0 mg/Kg) had no significantly difference, indicating that C-I20 supplementation did not improve the nutritional value of amino acid composition (**Table 3**). The mandarin fish fed 160 mg/Kg C-I20 had significantly higher total polyunsaturated fatty acids (∑PUFA) and eicosapentaenoic acid (EPA) + docosahexaenoic acid (DHA), but had significantly lower total short chain fatty acids (∑SFA) than the fish fed C-I20 (FM, 320, 80, 40 and 0 mg/Kg) (**Table 4**). The FM-fed and 160 mg/Kg C-I20-fed fish had statistically similar total monounsaturated fatty acids (∑MUFA), EPA + DHA, total n-3 polyunsaturated fatty acids (∑n-3), total n-6 polyunsaturated fatty acids (∑n-6) and ∑PUFA values, but had markedly higher ∑SFA (**Table 4**). The fish in FM group had significantly higher ∑EAA, ∑NEAA and ∑FAA values compared with the other groups (**Table 4**). In summary, 160 mg/Kg C-I20 supplementation partly improved the ∑PUFA (especially EPA + DHA) in muscle tissue of mandarin fish.

**Table 2:** Muscle composition of mandarin fish fed with the experimental diets.

Parameters (%)	Group					
	FM	320 mg/Kg	160 mg/Kg	80 mg/Kg	40 mg/Kg	Control
Moisture	77.21 ± 1.52^a^	77.58 ± 1.41^a^	78.25 ± 1.63^a^	79.58 ± 0.98^a^	77.68 ± 1.24^a^	78.55 ± 1.13^a^
Crude protein	19.24 ± 0.43^a^	19.03 ± 0.26^a^	18.88 ± 0.36^a^	19.16 ± 0.28^a^	18.79 ± 0.37^a^	18.93 ± 0.29^a^
Crude lipid	1.47 ± 0.28^a^	1.46 ± 0.35^a^	1.52 ± 0.32^a^	1.43 ± 0.24^a^	1.61 ± 0.48^a^	1.34 ± 0.52^a^
Ash	1.25 ± 0.05^a^	1.43 ± 0.11^a^	1.30 ± 0.14^a^	1.35 ± 0.21^a^	1.29 ± 0.08^a^	1.37 ± 0.13^a^

Values are presented as the means ± SD (n = 12). In the same row, values with the same letter superscripts represent no significant difference (P > 0.05).

**Table 3:** Amino acid contents in the muscle of mandarin fish fed with the experimental diets.

Item (% dry basis)	Treatment					
	FM	320	160	80	40	0
Arg	4.54 ± 0.12	4.02 ± 0.05	4.38 ± 0.13	4.18 ± 0.05	4.17 ± 0.09	4.21 ± 0.06
His	3.66 ± 0.05	3.40 ± 0.06	3.57 ± 0.10	3.36 ± 0.10	3.36 ± 0.02	3.40 ± 0.11
Val	3.90 ± 0.05	3.65 ± 0.08	3.84 ± 0.06	3.56 ± 0.07	3.64 ± 0.10	3.59 ± 0.09
Phe	3.78 ± 0.05	3.59 ± 0.09	3.79 ± 0.09	3.58 ± 0.06	3.57 ± 0.19	3.61 ± 0.15
Leu	6.67 ± 0.08	6.39 ± 0.14	6.66 ± 0.08	6.43 ± 0.09	6.35 ± 0.06	6.28 ± 0.08
Ile	3.52 ± 0.05	3.22 ± 0.07	3.45 ± 0.09	3.22 ± 0.15	3.28 ± 0.06	3.24 ± 0.20
Thr	4.35 ± 0.52	3.71 ± 0.06	3.81 ± 0.06	3.69 ± 0.26	3.64 ± 0.09	3.66 ± 0.14
Met	2.36 ± 0.06	2.13 ± 0.10	2.32 ± 0.07	2.15 ± 0.21	2.08 ± 0.08	2.03 ± 0.09
Lys	7.53 ± 0.08	7.27 ± 0.09	7.49 ± 0.06	7.22 ± 0.13	7.23 ± 0.07	7.30 ± 0.10
ΣEAA^A^	40.32 ± 0.53^b^	37.55 ± 0.25^a^	38.34 ± 0.57^a^	37.34 ± 0.37^a^	37.32 ± 0.36^a^	37.32 ± 0.11^a^
Asp	7.42 ± 0.10	7.13 ± 0.16	7.23 ± 0.10	7.12 ± 0.18	7.03 ± 0.24	6.97 ± 0.21
Ser	3.31 ± 0.09	2.88 ± 0.21	2.94 ± 0.09	2.89 ± 0.15	2.79 ± 0.14	2.91 ± 0.15
Glu	13.02 ± 0.68	11.40 ± 0.16	11.81 ± 0.31	11.39 ± 0.11	11.40 ± 0.33	11.66 ± 0.42
Ala	4.85 ± 0.10	4.47 ± 0.13	4.68 ± 0.07	4.44 ± 0.23	4.54 ± 0.19	4.40 ± 0.10
Gly	3.86 ± 0.11	3.69 ± 0.10	3.75 ± 0.10	3.69 ± 0.07	3.65 ± 0.11	3.72 ± 0.10
Tyr	2.37 ± 0.18	1.99 ± 0.14	2.15 ± 0.16	2.06 ± 0.10	2.09 ± 0.19	1.99 ± 0.13
ΣNEAA^B^	34.82 ± 1.15^b^	31.56 ± 0.21^a^	32.53 ± 0.72^a^	31.58 ± 0.26^a^	31.50 ± 0.93^a^	31.66 ± 0.84^a^
ΣFAA^C^	39.84 ± 0.99^b^	36.74 ± 0.45^a^	37.78 ± 0.81^a^	36.45 ± 0.50^a^	36.45 ± 1.26^a^	36.56 ± 0.75^a^

All values are presented as mean ± SD (twelve fish flesh samples per group were collected and four flesh samples were mixed as a pooled sample. A total of three pooled samples for assays, n = 3). In the same row, values with the same letter superscripts represent no significant difference (P > 0.05), while with different letter superscripts represent significant differences (P< 0.05).

^A^EAA is the sum of essential amino acids, which includes Arg, His, Val, Phe, Leu, Ile, Thr, Met and Lys.

^B^NEAA is the sum of non essential amino acids, which includes Asp, Ser, Glu, Ala, Gly and Tyr.

^C^FAA is the sum of flavor amino acids, which includes Glu, Ala, Asp, Gly, Phe, Arg and Tyr.

**Table 4:** Fatty acid composition in muscle of mandarin fish fed with the experimental diets.

Parameters (%)	Group					
	FM	320 mg/Kg	160 mg/Kg	80 mg/Kg	40 mg/Kg	Control
∑SFA^1^	36.54 ± 1.15^b^	38.55 ± 0.65^b^	34.27 ± 0.74^a^	40.29 ± 0.61^c^	40.33 ± 1.24^c^	38.75 ± 1.58^bc^
∑MUFA^2^	20.39 ± 0.61^a^	21.25 ± 0.38^a^	20.87 ± 1.15^a^	22.24 ± 0.73^a^	21.07 ± 0.42^a^	21.76 ± 0.78^a^
EPA + DHA	1.58 ± 0.13^c^	1.12 ± 0.11^b^	1.45 ± 0.08^c^	0.96 ± 0.07^a^	0.83 ± 0.14^a^	0.93 ± 0.07^a^
∑n-3^3^	18.25 ± 0.73^a^	18.83 ± 0.65^a^	19.87 ± 0.37^a^	17.38 ± 1.36^a^	18.54 ± 1.02^a^	18.32 ± 0.63^a^
∑n-6^4^	23.62 ± 0.78^b^	21.47 ± 1.07^ab^	23.35 ± 0.85^b^	19.47 ± 1.18^a^	19.09 ± 0.47^a^	20.67 ± 0.96^a^
∑PUFA^5^	41.21 ± 1.37^c^	38.24 ± 1.43^b^	42.48 ± 2.28^c^	36.25 ± 0.87^a^	35.24 ± 1.36^a^	37.09 ± 2.31^a^

All values are presented as mean ± SD (twelve fish flesh samples per group were collected and four flesh samples were mixed as a pooled sample. A total of three pooled samples for assays, n = 3). In the same row, values with the same letter superscripts represent no significant difference (P > 0.05), while with different letter superscripts represent significant differences (P< 0.05).

^1^∑SFA is the total of saturated fatty acids, which includes C14: 0, C15: 0, C16: 0, C17: 0, C18: 0, C22: 0 and C23: 0.

^2^∑MUFA is the total of monounsaturated fatty acids, which includes C14: 1, C15: 1, C16: 1, C17: 1, C18: 1, C20: 1 and C22: 1.

^3^∑n-3 is the total of n-3 polyunsaturated fatty acids.

^4^∑n-6 is the total of n-6 polyunsaturated fatty acids.

^5^∑PUFA is the total of polyunsaturated fatty acids, which includes C18: 2 n-6 and C18: 3 n-6, C18: 3 n-3, C20: 3 n-6, C20: 3 n-3, C20: 4 n-6, C20: 5 n-3 (EPA) and C22: 6 n-3 (DHA).

## 4 Discussion

Aquaculture development has recently been negatively impacted by declining output and rising price of FM (24). As a response, more people are becoming interested in using plant protein as feedstock, which is a potential strategy for fostering the industry’s sustainable growth (4). However, replacement of FM with plant protein was found to negatively affect the feeding rate and growth performance of rainbow trout (25), Atlantic salmon (26) and turbot (27). In addition, replacement of FM with plant protein was found to affect the muscle texture and nutritive value of Yellow River carp (28) and Nile tilapia (5). Hence, it is of great significance to reduce the side effects of plant protein on fillet quality traits for the development of the aquaculture industry. In the present study, C-I20 was supplemented in the diet of FM partially replaced by soybean meal to explore mucosal barrier status, internal organs health status, growth performance, body composition, muscle quality, amino acid composition and fatty acid composition of oral C-I20 administration.

CMCS drug delivery nanotechnology provides a novel method to overcome the disadvantage of using peptide-based agents, especially in avoiding the digestion by enzymes and controlling the release (29, 30). CMCS has abundant functional groups (–OH, –COOH and –NH) to adsorb AMPs (31). We coupled CMCS with gcIFN-20H to prepare uniformly spherical nanoparticles (C-I20) with gcIFN-20H on the surface. The aggregations of gcIFN-20H on the surface of C-I20 nanoparticles generate a steric barrier against degradation by adverse circumstances, which is one of the principles by which C-I20 nanoparticles can resist degradation (22). In our experiment, the free gcIFN-20H were readily degraded in the stomach by pepsin, making it difficult for gcIFN-20H to be carried into the intestine to function. C-I20 effectively resisted the degradation of pepsin, indicating that C-I20 nanoparticles had a better chance to function in the intestine than free gcIFN-20H. It is worth noting that the gastric environment (acid and rich in pepsin) accelerates the release rate of C-I20, thus accelerating the degradation of gcIFN-20H, suggesting that we need to increase the applied dosage of C-I20 in an adverse environment.

In this study, supplementary doses below 160 mg/kg had limited positive effects on mandarin fish. The low dose of C-I20 failed to have a positive effect due to the addition of a small amount of C-I20 in the low dose group and the degradation of the gastric environment. The fish in the 160 mg/kg C-I20 group outperformed the other oral C-I20 groups in terms of FBW, WGR, FCR and crude protein content. The reason for this result was that the fish fed with 160 mg/kg C-I20 had an appropriate mucosal barrier and organs to promote growth. The intestinal barrier could effectively prevent anti-nutritional factors from damaging the intestinal digestive system and promote the growth of fish (32). The thickness of mucin and the number of goblet cells in the 80, 40 and 0 mg/kg groups were significantly reduced in comparison to the 320 and 160 mg/kg groups. The relative decrease in mucin and goblet cells in the low-dose group may be due to the long-term damage to the intestinal mucosa caused by anti-nutritional factors in plant proteins (4). The thickening of mucin in the high-dose feeding group can resist the damage of irritants to maintain the normal function of the intestinal tract. However, a mucin layer that is too thick may affect the intestinal digestion and absorption function (33). This may be the reason that the application effect of 320 mg/kg C-I20 treatment was not superior to that of 160 mg/kg C-I20 treatment. Therefore, 160 mg/kg C-I20 treatments with similar mucin thickness to FM treatment might be a suitable feeding strategy. Due to the limited research on the regulation mechanism of intestinal barrier of mandarin fish, the molecular mechanism of C-I20 acting on intestinal mucosa needs to be further explored. Notably, the mucosal barriers are sufficient to fend off various types of antinutritional factors of plant protein sources (7). Therefore, C-I20 is likely to help fish adapt to a variety of plant protein sources. In this study, mandarin fish fed diets with 160 mg/kg C-I20 showed increased villus height. Intestine is the place for digestion and absorption of nutrients, and its morphology can directly reflect intestinal function (34). In general, larger villus dimensions indicate a larger contact area, which may promote the intestinal absorption function of hosts (35). The increased intestinal cross-sectional area and muscularis thickness is positively correlated with the body weight and body growth of fish in previous studies (35). The increased intestinal cross-sectional area and muscularis thickness were also observed in two fast growing groups (FM and 160 mg/kg). As a whole, intestinal morphology and weight gain of fish in 160 mg/kg group formed a benign interaction, resulting in the common improvement of the two indicators.

A previous study that added SM to the diet of Atlantic salmon found that excessive amounts of FM substitution with plant protein could harm the liver system (36). This may be caused by the direct damage to liver cells caused by antinutrition factors entering the blood. As can be observed, groups receiving FM, 320 mg/kg and 160 mg/kg inflict less liver damage than the other groups. Vacuole formation in the FM, 320 mg/kg and 160 mg/kg groups was less than that in the 80 mg/kg, 40 mg/kg and control groups. The reason for this result may be that the FM, 320 mg/kg and 160 mg/kg groups had an appropriate mucosal barrier to resist the invasion of anti-nutritional factors. ALT, ALP and AST concentrations in serum can rise as a result of liver injury (37). The activity of ALT, ALP and AST in groups 80 mg/Kg, 40 mg/Kg and control was significantly higher than that in the other groups. The immediate cause for this result is liver tissue produces a large number of cell vacuoles and releases a large number of ALT, ALP and AST into serum. Surprisingly, the feeding treatment of 320 mg/Kg and 160 mg/Kg C-I20 can greatly lessen liver damage and maintain normal liver function. The feeding treatment of 320 mg/kg and 160 mg/kg C-I20 significantly improved the health status of tissues (liver, trunk kidney, head kidney and spleen) and hardly suffered from inflammation injury. However, only histological change can be observed by tissue section examination. The reason for the protective effect of C-I20 needs to be analyzed at the molecular level.

Morphology traits such as a total number of fibers and myofiber diameter are major determinant factors of muscle mass as well as meat quality (38). According to a prior study, myofiber hypertrophy and hyperplasia, which increase in myofiber diameter and quantity, respectively, are the key determinants of myofiber growth (39). The myofiber diameters in fish fed 160 mg/kg C-I20 and FM diets-feed were substantially higher than those in the other groups in our experiment. The trend of this result is consistent with the WGR, suggesting that the increase in body weight is partly due to the thickening of muscle fiber diameter. Due to the high muscle fiber diameter of group FM and 160 mg/Kg, group FM and 160 mg/Kg had considerably fewer muscle fibers per unit area in the same visual field as the other groups. Physical performance indicators such as hardness, springiness and chewiness are important indexes in evaluating fillet quality (40). In comparison to the other groups, the 160 mg/Kg group had much less hardness and chewiness while having significantly more springiness. Some fish studies have pointed out that muscle hardness is also negatively correlated with the myofiber diameter or nutritional status (41), which are consistent with our results. The elevated inflammation also enhanced the hardness of farmed animal muscle (8). Oxidative stress caused by inflammation might reduce the synthesis of new collagen in skeletal muscle fibroblasts and decrease the solubility of collagen, thereby increasing meat toughness and inhibiting muscle fiber thickening (42). An appropriate amount of C-I20 has the ability to alleviate the tissue inflammation caused by soybean meal and resist oxidative stress, so the muscle hardness of mandarin fish in the 160 mg/Kg group decreased and the muscle fiber thickened. These results indicate that adding a reasonable concentration of C-I20 can significantly increase muscle fiber diameter and maintain physical performance of fillets, thus improving fillet quality of mandarin fish.

There were no significant differences in moisture, crude protein, crude lipid and ash of white muscle in the six groups. These results indicate that C-I20 has no negative effect on moisture, crude protein, crude lipid and ash of white muscle. Apart from muscle composition, fatty acids and amino acids have a great influence on the nutritional value and flavor of fish fillets (23). Our present study indicated that there was no significant change in the ratio of ΣEAA, ΣNEAA and ΣFAA in C-I20-fed group compared with the control group, while those of FM group were significantly higher than those of each group. The content of amino acids in muscle of each group was basically consistent with that in feed. The main factor affecting the proportion of amino acids in muscle is that the proportion of amino acids in feed is not the addition of C-I20. Fish is very unique, which have significant PUFA biosynthetic capacity, providing the main source of PUFA in human diets (43). Previous study indicated that fish oil containing polyunsaturated fatty acids (such as EPA and DHA) has beneficial health functions in animal model and human (43). Interestingly, our research indicated that the contents of ΣPUFA (including ∑n-6 and EPA + DHA) increased in the 160 mg/kg C-I20-fed fish than the other C-I20-fed (320, 80, 40, 0 mg/kg) fish. Polyunsaturated fatty acids (PUFAs) are important structural components of the cell membrane (44). Compared with the 160 mg/kg and 320 mg/kg groups, the 80 mg/kg, 40 mg/kg and control groups showed more severe tissue inflammatory lesions. Mandarin fish in the 80 mg/kg, 40 mg/kg and control groups needed to consume more PUFAs to repair the cell membrane, resulting in a decrease in the deposition ratio of PUFAs in muscle. The reduced level of ∑SFA in 160 mg/kg C-I20-fed fish may reflect its use as an energy supply because lipids are the main energy source for growth and development. In addition, the liver is responsible for the metabolism, synthesis, storage and redistribution of nutrients such as fats, carbohydrates and vitamins (45), and liver injury is also an important reason for the difference in fatty acid composition in the muscle of each treatment group (46). However, the molecular mechanism of C-I20 affecting muscle fiber changes and fatty acid content has not been further elucidated. More work is needed to gain insight into the effect of C-I20 on fatty acid composition. Compared to ΣPUFA, ΣSFA have a lower cholesterol-reducing ability, potentially increasing the prevalence of coronary heart disease and obesity in humans (47). These results also show that eating this fish fillet has many benefits for human health. In general, 160 mg/kg C-I20 supplementation significantly increased the ΣPUFA of fillets, especially DHA + EPA, leading to improve the nutritive value of fillets.

## 5 Conclusion

Our researches demonstrated, for the first time, that dietary AMP preparation supplementation efficiently offsets the detrimental effects of SM replacement on growth performance and fillet quality traits in aquaculture. In this study, C-I20 nanoparticles exhibit excellent feature of sustained-release and are able to effectively prevent pepsin degradation, which makes them efficiently transport to the intestinal tract to perform functions. Dietary 160 mg/Kg C-I20 supplementation significantly improved the growth performance. 160 mg/Kg C-I20 treatment effectively improves intestinal mucosal barrier, intestinal morphology and reduces multi-type tissue (liver, trunk kidney, head kidney and spleen) injury. 160 mg/Kg C-I20 treatment prevents the reduction in myofiber diameter and change in hardness, springiness and chewiness, while effectively improves polyunsaturated fatty acids (especially DHA + EPA) in fillet. Therefore, dietary C-I20 in a reasonable concentration supplementation is a prospectively novel strategy for improving growth performance and fillet quality traits in aquaculture.

## Data availability statement

The original contributions presented in the study are included in the article/**Supplementary Material**. Further inquiries can be directed to the corresponding author.

## Ethics statement

All procedures of animal experiments were approved by the Ethical Committee on Animal Research at Huazhong Agricultural University (ID Number: HZAUFI-2022-0031).

## Author contributions

JS and XH conceived and designed the experiments and wrote the manuscript. XH, QZ, JC, GY, SH, and CY performed the experiments and analyzed the data. JS, YZ, XL, and SH revised the manuscript critically. All authors contributed to the article and approved the submitted version.
