# Supplementary material for: Nanopeptide C-I20 as a novel feed additive effectively alleviates detrimental impacts of soybean meal on mandarin fish by improving the intestinal mucosal barrier
Source: Front Immunol. 2023 Jun 26;14:1197767. doi: 10.3389/fimmu.2023.1197767 (PMC10331600; doi:10.3389/fimmu.2023.1197767)
Supplement: Supplementary file 1 [file DataSheet_1.docx]

# Supplementary Tables

## Table S1. Composition of diets added with different levels of nanopeptide C-I20.

|  | Experimental diets | | | | | |
| --- | --- | --- | --- | --- | --- | --- |
| Group (mg/Kg) | FM | 320 | 160 | 80 | 40 | Control |
| Ingredients (g kg^-1^ diet) | | | | | | |
| Fish meal^1^ | 480.00 | 300.00 | 300.00 | 300.00 | 300.00 | 300.00 |
| Casein^2^ | 150.00 | 150.00 | 150.00 | 150.00 | 150.00 | 150.00 |
| Soybean meal^3^ | 0.00 | 180.00 | 180.00 | 180.00 | 180.00 | 180.00 |
| Fish oil | 80.00 | 80.00 | 80.00 | 80.00 | 80.00 | 80.00 |
| Ca (H_2_PO_4_)_2_ | 17.00 | 17.00 | 17.00 | 17.00 | 17.00 | 17.00 |
| Microcrystalline cellulose | 30.00 | 30.00 | 30.00 | 30.00 | 30.00 | 30.00 |
| Carboxymethylcellulose sodium | 183.00 | 182.68 | 182.84 | 182.92 | 182.96 | 183.00 |
| Mineral mix^4^ | 20.00 | 20.00 | 20.00 | 20.00 | 20.00 | 20.00 |
| Vitamin mix^5^ | 20.00 | 20.00 | 20.00 | 20.00 | 20.00 | 20.00 |
| C-I20 | 0.00 | 0.32 | 0.16 | 0.08 | 0.04 | 0.00 |
| Total | 1000.00 | 1000.00 | 1000.00 | 1000.00 | 1000.00 | 1000.00 |
| Proximate composition | | | | | | |
| Crude protein (%) | 42.1 | 42.7 | 41.9 | 42.3 | 42.2 | 46.2 |
| Crude lipid (%) | 8.5 | 8.4 | 8.7 | 8.8 | 8.3 | 8.1 |
| Ash (%) | 16.2 | 14.3 | 14.6 | 14.2 | 14.5 | 14.6 |

^1^ Crude protein and carbohydrate content of fish meal was 67% and 7%, respectively.

^2^ Crude protein and crude lipid content of casein was 89% and 0.8%, respectively.

^3^ Crude protein and crude lipid content of soybean meal was 42% and 2.1%, respectively.

^4^ Mineral premix (per kg of diet): MnSO_4_, 10 mg; MgSO_4_, 10 mg; KCl, 95 mg; NaCl, 165 mg; ZnSO_4_, 20 mg; KI, 1 mg; CuSO_4_,12.5 mg; FeSO_4_, 105 mg; Na_2_SeO_3_, 0.1 mg; Co, 1.5 mg.

^5^ Vitamin premix (per kg of diet): vitamin A, 2000 IU; vitamin B_1_ (thiamin), 5 mg; vitamin B_2_ (riboflavin), 5 mg; vitamin B_6_, 5 mg; vitamin B_12_, 0.025 mg; vitamin D_3_, 1200 IU; vitamin E 21 mg; vitamin K_3_ 2.5 mg; folic acid, 1.3 mg; biotin, 0.05 mg; pantothenic acid calcium, 20 mg; inositol, 60 mg; ascorbic acid (35%), 110 mg; niacinamide, 25 mg.

## Table S2 Amino acid compositions of the tested diets.

| Item (% dry basis) | Treatment | | | | | |
| --- | --- | --- | --- | --- | --- | --- |
|  | FM | 320 | 160 | 80 | 40 | 0 |
| Arg | 4.45 ± 0.01 | 3.62 ± 0.03 | 3.69 ± 0.04 | 3.73 ± 0.05 | 3.61 ± 0.03 | 3.66 ± 0.02 |
| His | 1.99 ± 0.04 | 1.63 ± 0.02 | 1.59 ± 0.06 | 1.62 ± 0.04 | 1.67 ± 0.02 | 1.63 ± 0.04 |
| Val | 2.48 ± 0.03 | 2.03 ± 0.03 | 2.08 ± 0.04 | 2.04 ± 0.05 | 1.96 ± 0.04 | 2.05 ± 0.04 |
| Phe | 2.21 ± 0.02 | 1.87 ± 0.03 | 1.84 ± 0.06 | 1.83 ± 0.01 | 1.88 ± 0.07 | 1.82 ± 0.05 |
| Leu | 3.95 ± 0.02 | 3.38 ± 0.05 | 3.47 ± 0.07 | 3.42 ± 0.04 | 3.47 ± 0.02 | 3.51 ± 0.01 |
| Ile | 2.12 ± 0.03 | 1.80 ± 0.05 | 1.79 ± 0.02 | 1.82 ± 0.02 | 1.82 ± 0.00 | 1.78 ± 0.03 |
| Thr | 2.21 ± 0.04 | 1.69 ± 0.01 | 1.72 ± 0.01 | 1.75 ± 0.05 | 1.73 ± 0.05 | 1.69 ± 0.02 |
| Met | 1.40 ± 0.04 | 1.07 ± 0.02 | 1.08 ± 0.02 | 1.14 ± 0.01 | 1.15 ± 0.03 | 1.13 ± 0.03 |
| Lys | 3.82 ± 0.02 | 2.95 ± 0.00 | 2.84 ± 0.04 | 2.93 ± 0.01 | 2.92 ± 0.02 | 2.92 ± 0.02 |
| ΣEAA^A^ | 24.62 ± 0.26^b^ | 20.13 ± 0.38^a^ | 20.11 ± 0.48^a^ | 20.28 ± 0.32^a^ | 20.16 ± 0.27^a^ | 20.18 ± 0.33^a^ |
| Asp | 4.42 ± 0.06 | 3.76 ± 0.06 | 3.62 ± 0.04 | 3.66 ± 0.03 | 3.72 ± 0.04 | 3.73 ± 0.05 |
| Ser | 2.53 ± 0.04 | 2.12 ± 0.03 | 2.23 ± 0.02 | 2.15 ± 0.06 | 2.21 ± 0.03 | 2.17 ± 0.02 |
| Glu | 7.45 ± 0.02 | 6.72 ± 0.05 | 6.68 ± 0.14 | 6.75 ± 0.05 | 6.65 ± 0.07 | 6.70 ± 0.13 |
| Ala | 2.53 ± 0.06 | 1.93 ± 0.03 | 2.12 ± 0.01 | 2.08 ± 0.01 | 1.98 ± 0.02 | 2.03 ± 0.07 |
| Gly | 2.77 ± 0.01 | 2.10 ± 0.02 | 2.02 ± 0.01 | 1.95 ± 0.04 | 2.03 ± 0.05 | 1.91 ± 0.08 |
| Tyr | 2.03 ± 0.07 | 1.65 ± 0.05 | 1.63 ± 0.03 | 1.75 ± 0.04 | 1.68 ± 0.02 | 1.71 ± 0.01 |
| ΣNEAA^B^ | 21.70 ± 0.34^b^ | 18.33 ± 0.45^a^ | 18.32 ± 0.52^a^ | 18.42 ± 0.38^a^ | 18.24 ± 0.37^a^ | 18.26 ± 0.40^a^ |
| ΣFAA^C^ | 21.41 ± 0.18^b^ | 18.03 ± 0.14^a^ | 17.91 ± 0.17^a^ | 18.02 ± 0.11^a^ | 17.94 ± 0.13^a^ | 17.90 ± 0.24^a^ |

All values are presented as mean ± SD (n = 3). In the same row, values with the same letter superscripts represent no significant difference (P > 0.05), while with different letter superscripts represent significant differences (P < 0.05).

^A^ EAA is the sum of essential amino acids, which includes Arg, His, Val, Phe, Leu, Ile, Thr, Met and Lys.

^B^ NEAA is the sum of non essential amino acids, which includes Asp, Ser, Glu, Ala, Gly and Tyr.

^C^ FAA is the sum of flavor amino acids, which includes Glu, Ala, Asp, Gly, Phe, Arg and Tyr.

## Table S3 Fatty acid composition of the tested diets.

| Parameters  (%) | Group | | | | | |
| --- | --- | --- | --- | --- | --- | --- |
|  | FM | 320 mg/Kg | 160 mg/Kg | 80 mg/Kg | 40 mg/Kg | Control |
| ∑SFA^1^ | 38.52 ± 1.02^a^ | 36.78 ± 1.37^a^ | 37.24 ± 1.86^a^ | 36.55 ± 1.55^a^ | 37.87 ± 1.39^a^ | 38.58 ± 1.24^a^ |
| ∑MUFA^2^ | 20.25 ± 1.68^a^ | 20.74 ± 1.26^a^ | 20.58 ± 0.97^a^ | 21.21 ± 1.32^a^ | 20.36 ± 1.51^a^ | 21.13 ± 1.22^a^ |
| EPA + DHA | 11.28 ± 0.35^a^ | 10.85 ± 0.63^a^ | 10.88 ± 0.74^a^ | 11.13 ± 0.69^a^ | 10.92 ± 0.43^a^ | 11.18 ± 0.37^a^ |
| ∑n-3^3^ | 23.85 ± 0.67^a^ | 23.63 ± 0.73^a^ | 22.37 ± 0.94^a^ | 23.36 ± 0.51^a^ | 23.58 ± 0.46^a^ | 23.53 ± 0.57^a^ |
| ∑n-6^4^ | 18.76 ± 0.62^a^ | 18.85 ± 0.86^a^ | 18.58 ± 0.37^a^ | 19.93 ± 0.28^a^ | 19.65 ± 0.54^a^ | 19.73 ± 0.47^a^ |
| ∑PUFA^5^ | 42.16 ± 1.12^a^ | 42.78 ± 0.93^a^ | 43.24 ± 1.35^a^ | 43.57 ± 0.87^a^ | 42.54 ± 1.08^a^ | 43.31 ± 1.18^a^ |

All values are presented as mean ± SD (n = 3). In the same row, values with the same letter superscripts represent no significant difference (P > 0.05).

^1^ ∑SFA is the sum of saturated fatty acids, which includes C14: 0, C15: 0, C16: 0, C17: 0, C18: 0, C22: 0 and C23: 0.

^2^ ∑MUFA is the total of monounsaturated fatty acids, which includes C14: 1, C15: 1, C16: 1, C17: 1, C18: 1, C20: 1 and C22: 1.

^3^ ∑n-3 is the total of n-3 polyunsaturated fatty acids.

^4^ ∑n-6 is the total of n-6 polyunsaturated fatty acids.

^5^ ∑PUFA is the total of polyunsaturated fatty acids, which includes C18: 2 n-6 and C18: 3 n-6, C18: 3 n-3, C20: 3 n-6, C20: 3 n-3, C20: 4 n-6, C20: 5 n-3 (EPA) and C22: 6 n-3 (DHA).
