# Supplementary material for: Nanopeptide C-I20 as a novel feed additive effectively alleviates detrimental impacts of soybean meal on mandarin fish by improving the intestinal mucosal barrier
Source: Front Immunol. 2023 Jun 26;14:1197767. doi: 10.3389/fimmu.2023.1197767 (PMC10331600; doi:10.3389/fimmu.2023.1197767)
Supplement: Supplementary file 2 [file DataSheet_2.docx]

# Supplementary Figures

***
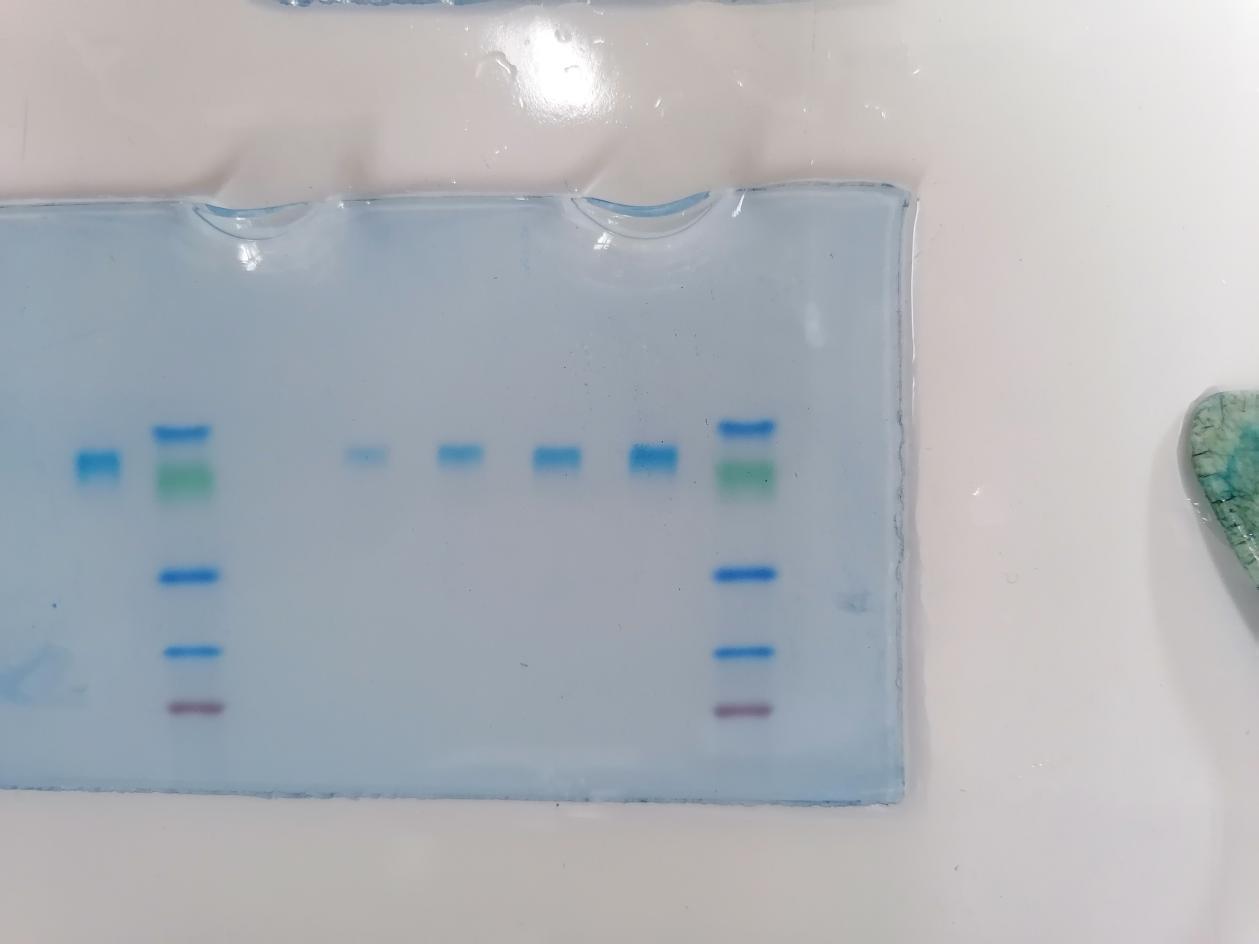
***

This SDS-PAGE assay result is the original image of Fig 1A.

*
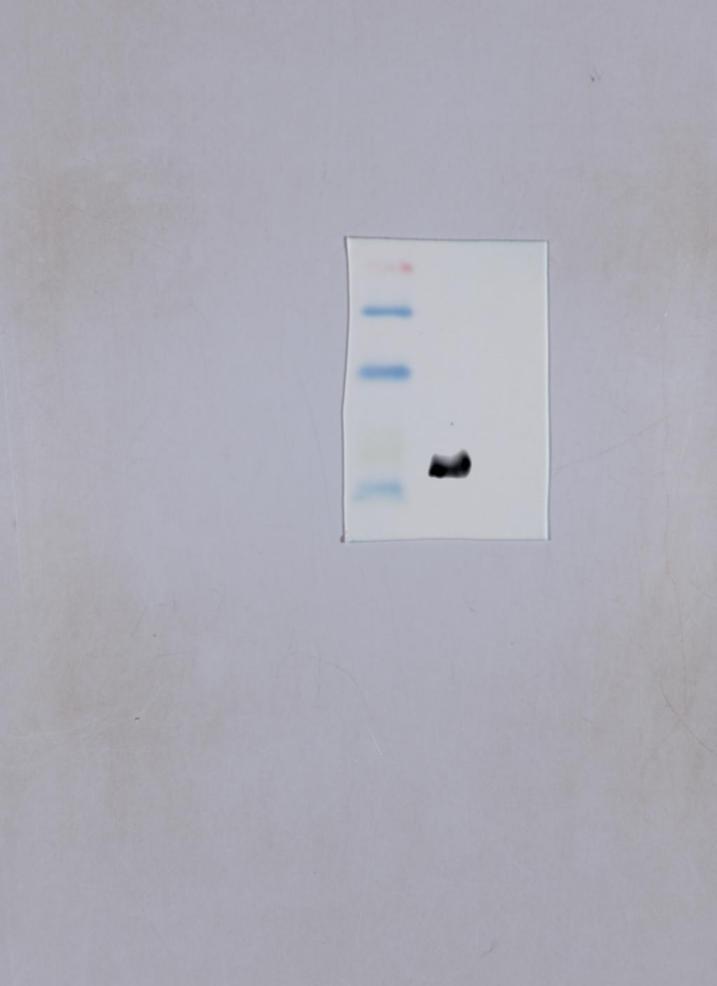
*

This WB assay result is the original image of Fig 1B.

*
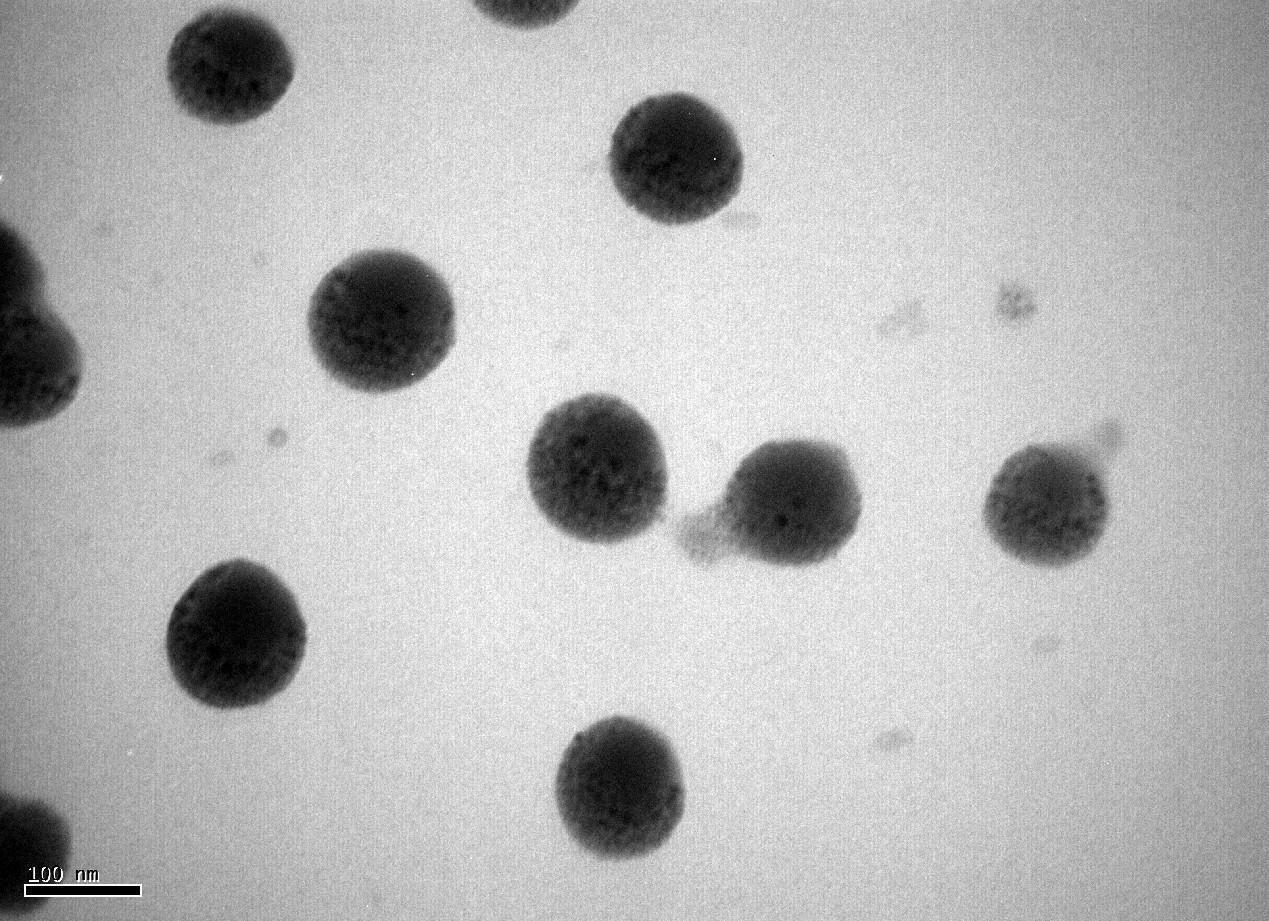
*

This transmission electron microscope assay result is the original image of Fig 1C.

*
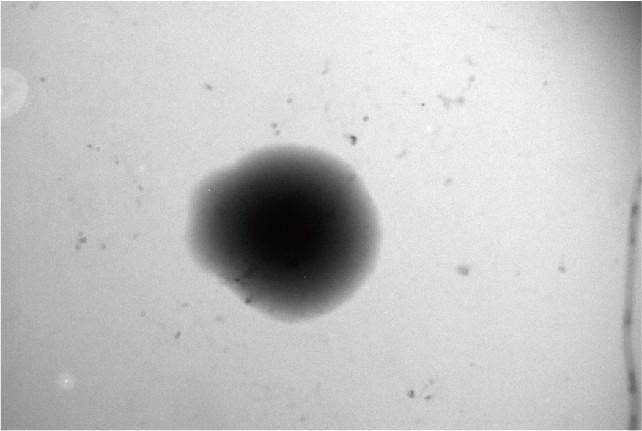
*

This transmission electron microscope assay result is the original image of Fig 1D.

*
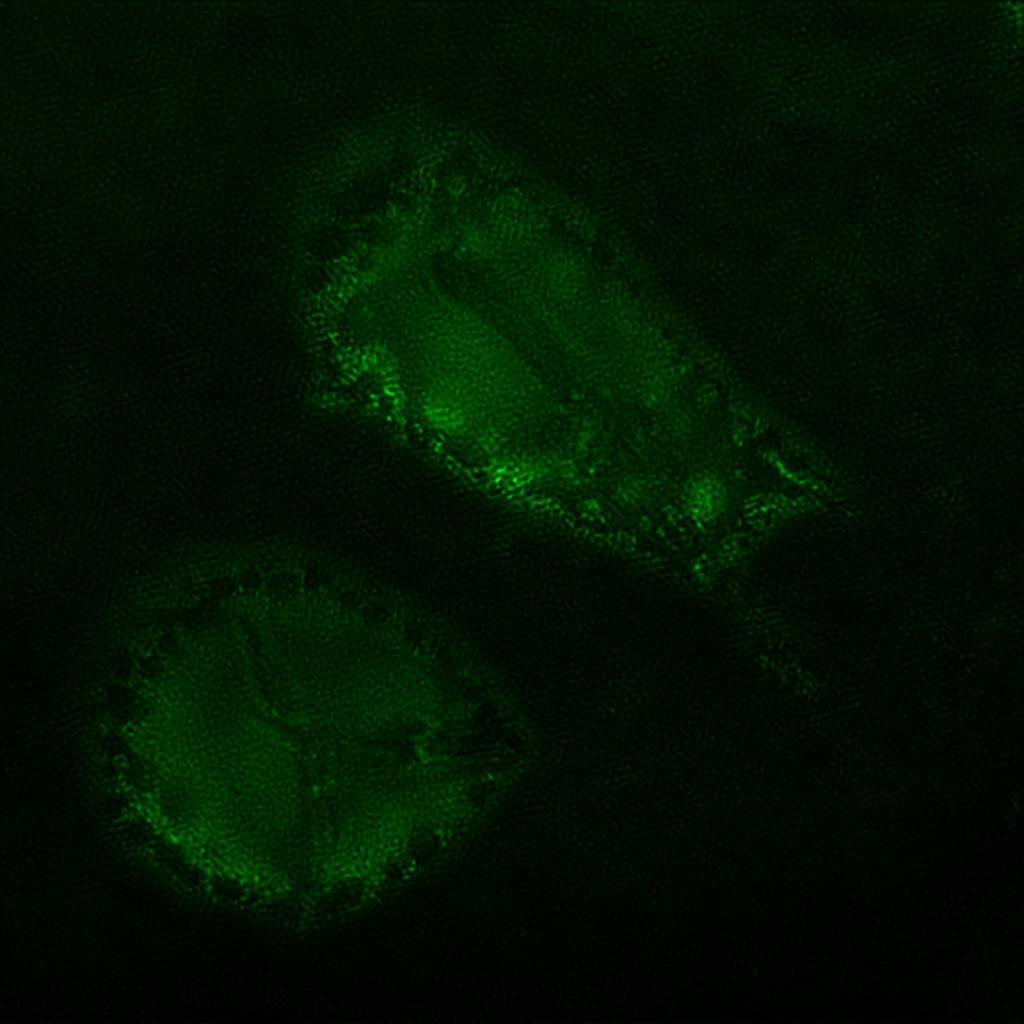
*

This fluorescence microscope assay result is the original image of Fig 1E.

*
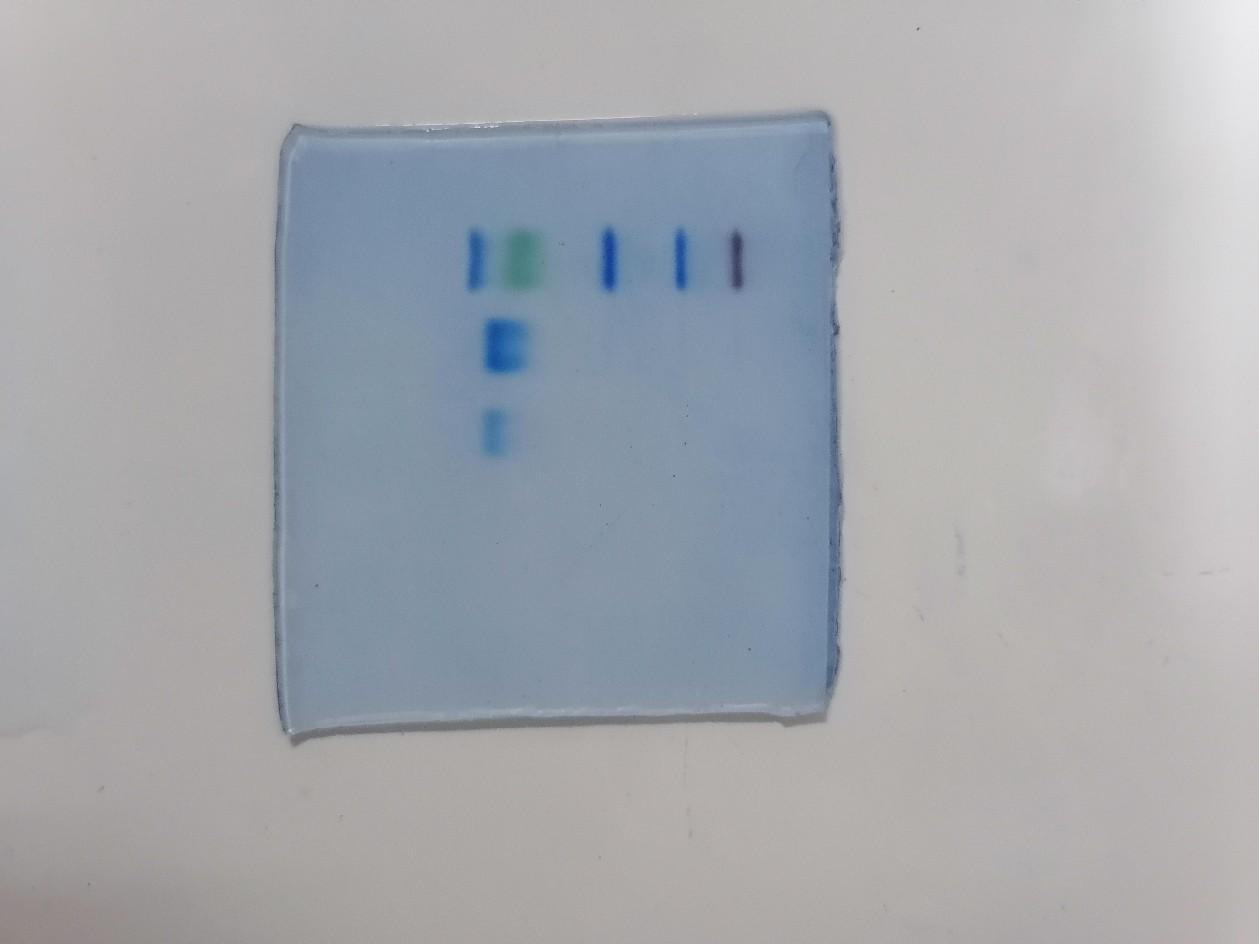
*

This SDS-PAGE assay result is the original image of Fig 1J.


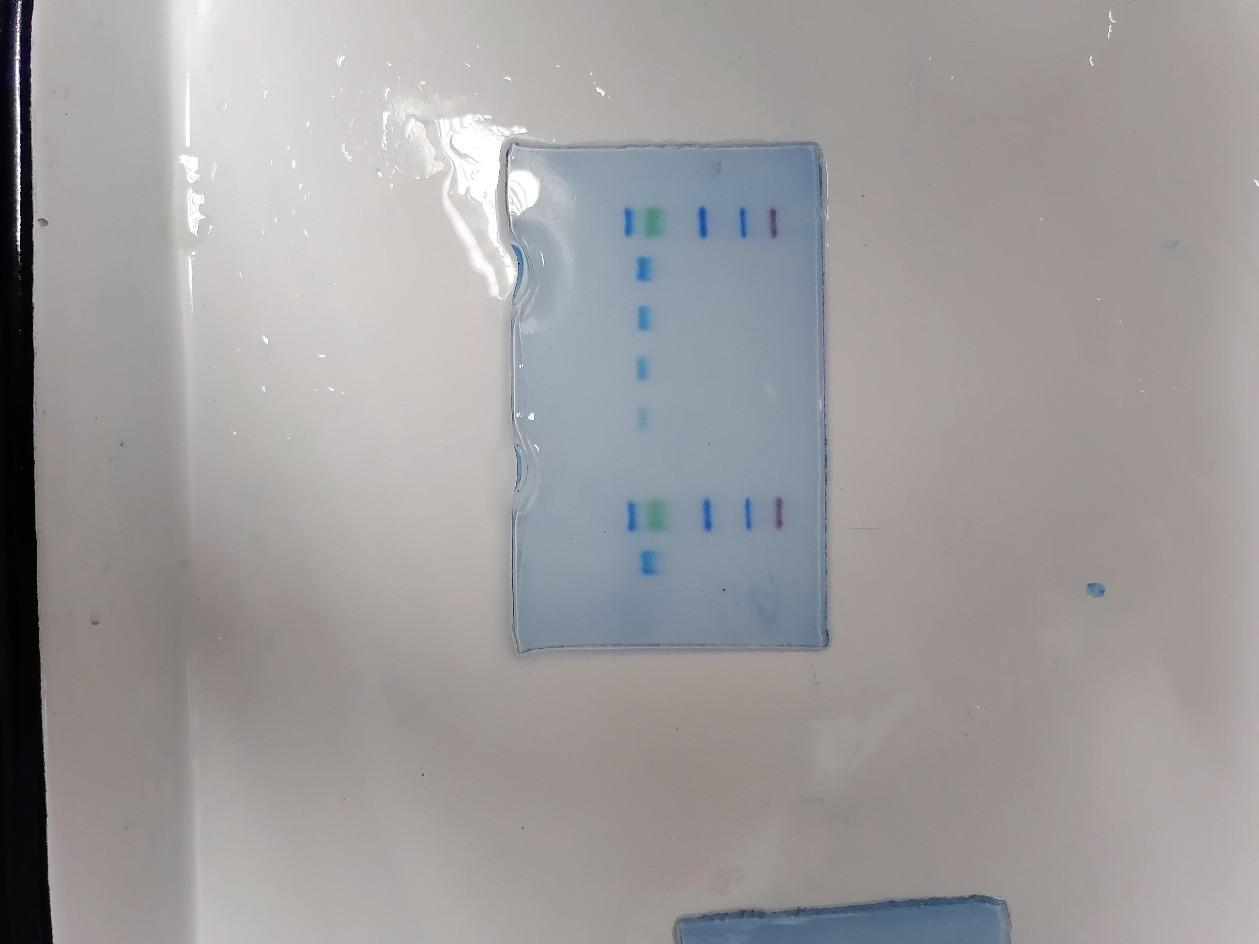


This SDS-PAGE assay result is the original image of Fig 1K.


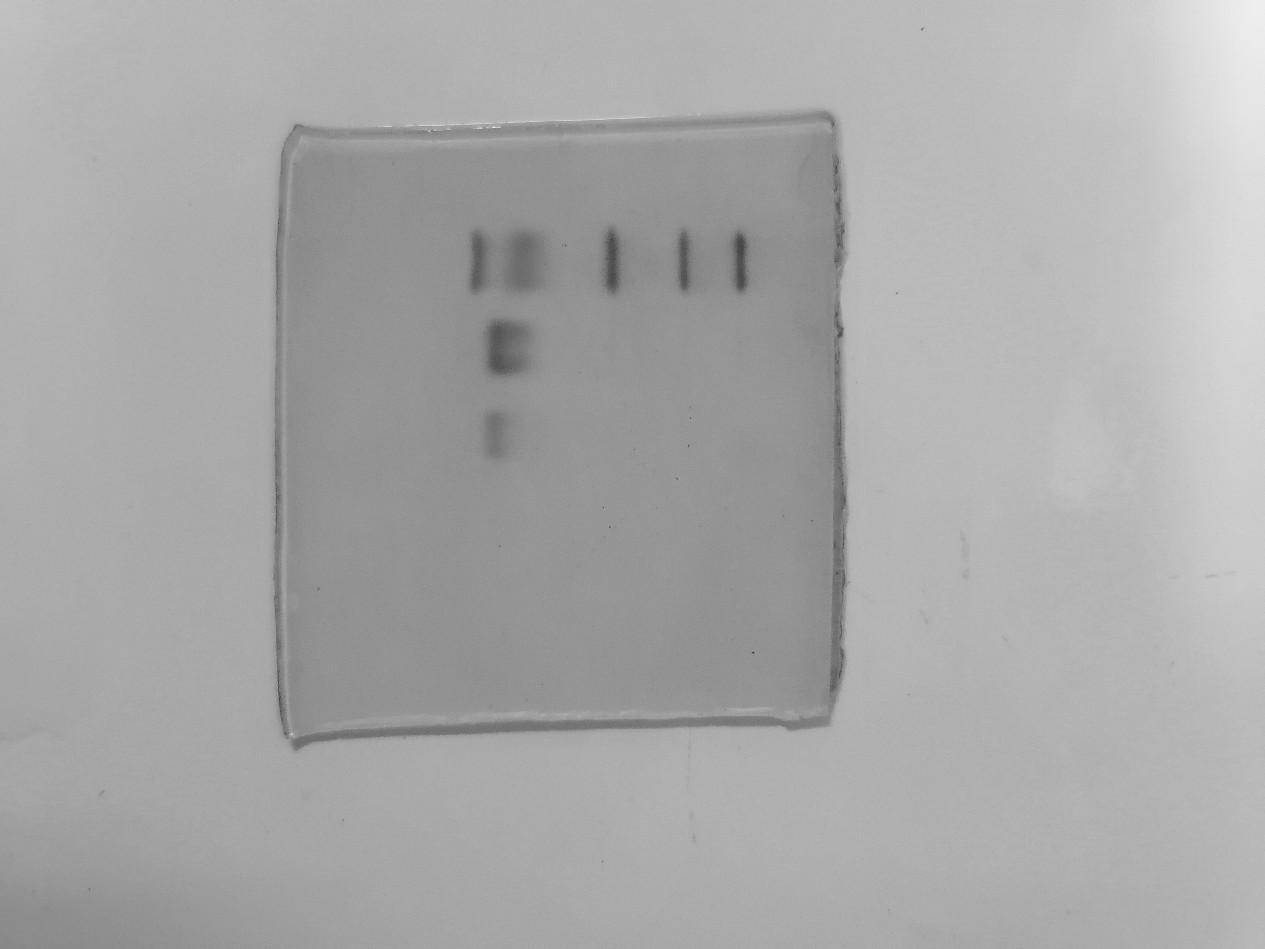


This SDS-PAGE gray value assay result is the original image of Fig 1L.


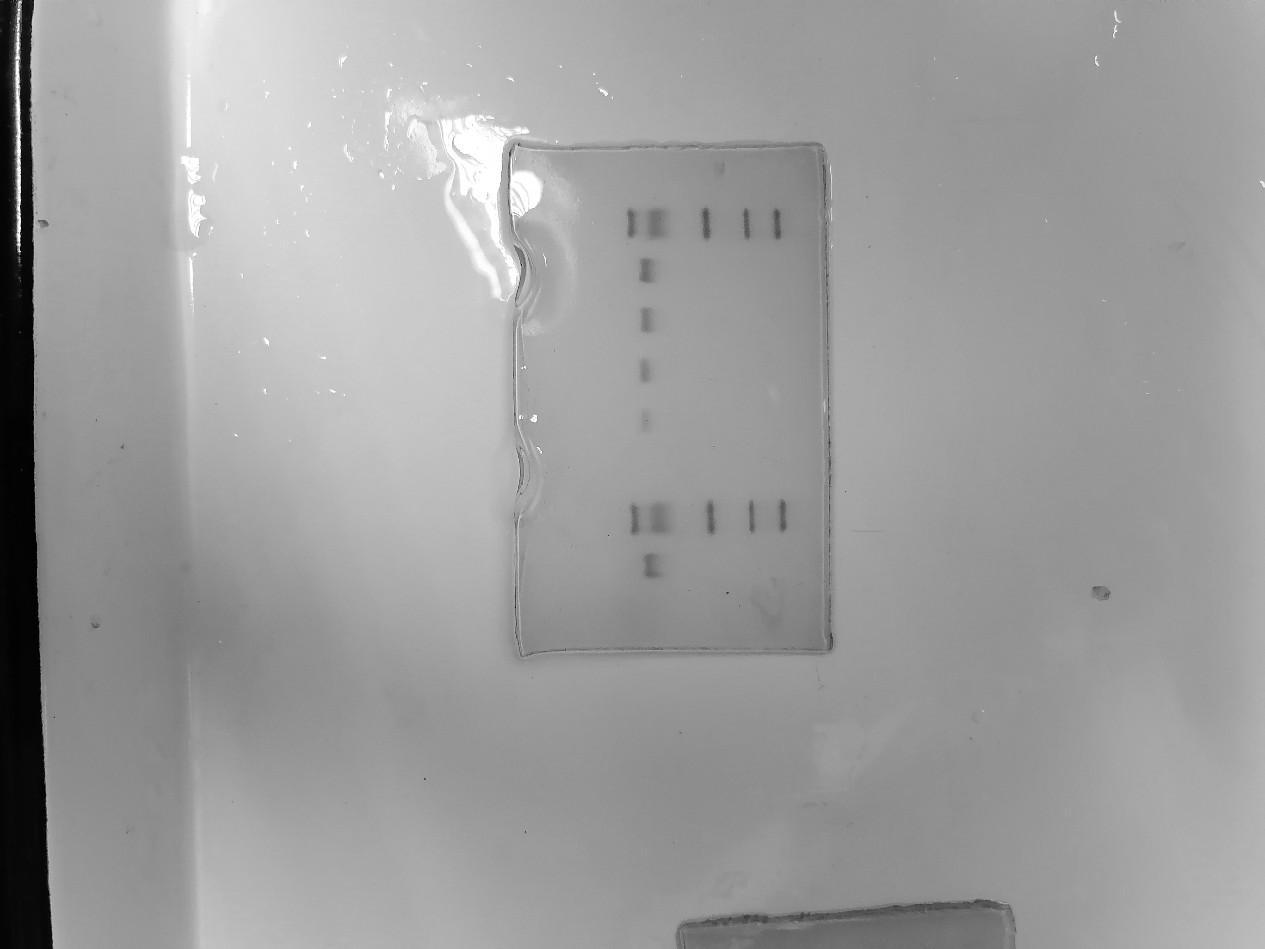


This SDS-PAGE gray value assay result is the original image of Fig 1M.
